# Pesticide Residues and Bees – A Risk Assessment

**DOI:** 10.1371/journal.pone.0094482

**Published:** 2014-04-09

**Authors:** Francisco Sanchez-Bayo, Koichi Goka

**Affiliations:** 1 Faculty of Agriculture and Environment, The University of Sydney, Eveleigh, New South Wales, Australia; 2 National Institute for Environmental Sciences, Tsukuba, Ibaraki, Japan; Federal University of Viçosa, Brazil

## Abstract

Bees are essential pollinators of many plants in natural ecosystems and agricultural crops alike. In recent years the decline and disappearance of bee species in the wild and the collapse of honey bee colonies have concerned ecologists and apiculturalists, who search for causes and solutions to this problem. Whilst biological factors such as viral diseases, mite and parasite infections are undoubtedly involved, it is also evident that pesticides applied to agricultural crops have a negative impact on bees. Most risk assessments have focused on direct acute exposure of bees to agrochemicals from spray drift. However, the large number of pesticide residues found in pollen and honey demand a thorough evaluation of all residual compounds so as to identify those of highest risk to bees. Using data from recent residue surveys and toxicity of pesticides to honey and bumble bees, a comprehensive evaluation of risks under current exposure conditions is presented here. Standard risk assessments are complemented with new approaches that take into account time-cumulative effects over time, especially with dietary exposures. Whilst overall risks appear to be low, our analysis indicates that residues of pyrethroid and neonicotinoid insecticides pose the highest risk by contact exposure of bees with contaminated pollen. However, the synergism of ergosterol inhibiting fungicides with those two classes of insecticides results in much higher risks in spite of the low prevalence of their combined residues. Risks by ingestion of contaminated pollen and honey are of some concern for systemic insecticides, particularly imidacloprid and thiamethoxam, chlorpyrifos and the mixtures of cyhalothrin and ergosterol inhibiting fungicides. More attention should be paid to specific residue mixtures that may result in synergistic toxicity to bees.

## Introduction

Growing concern about the impact of pesticides on pollinators is reflected in the enormous literature on the topic in the past few years [1]. In response to this concern, considerable amounts of new data on toxic effects of pesticides on wild bees, in particular bumble bees, have been obtained from laboratory and semi-field experiments [2], [3].

A number of reviews on the topic have highlighted the importance of bees as natural pollinators not only for our crops but also for wildflowers and plants of forests and tropical ecosystems [4], [5]. That is why the current declining trend of pollinators is worrying [6]. For example, it has been estimated that without bees, some 60 species of crop plants would fail to produce fruit [7]; the economic consequences of this impact are obvious. Importation of bumble bees to make up for the losses of pollinators in the areas affected not only does not solve the issue but also creates more problems by exporting parasites to other regions or countries [8], [9] or competing with native species [10].

Of particular importance is the collapse of honey bee (*Apis mellifera*) colonies (CCD) in America and other developed countries, because they provide honey and wax commodities to our society. Attempts to explain the CCD have focussed on two main fronts: i) biological diseases, which includes virus [11] *Nosema* infections [12], parasites such as mites [13], [14] and hive beetles [15]; and ii) pesticides, including not only insecticides and acaricides but also fungicides and herbicides [16], [17]. Naturally, low levels of pesticides may act as stressors that make bees more prone to biological infections [3], [18], [19]. Among the pesticides, newly developed systemic insecticides such as fipronil and neonicotinoids have been targeted as the main culprits involved in the collapses since they were launched to the market in the mid-1990s [20], [21], [22], [23].

Biological factors have been responsible for many of the problems that beekeepers have with their bee hives [24], but they are unlikely to be the main cause of disappearance of a number of wild bee species, or the decline of bumble bees in North America and Europe in recent years [12], [25]. Although there are scant data on bee populations from other parts of the world to make a proper evaluation, the fact that bee declines have been observed in countries that have a long history of using pesticides in agriculture points to these agrochemicals as one of the important factors underlying wild bee and honey bee colony losses. To resolve this issue, several surveys have been carried out in recent years in North America [26], [27], [28], France [29], [30], Spain [31] and India [32] among others, to find out the amounts and prevalence of pesticide residues present in pollen, honey, wax and other matrices of the bee hives (e.g. combs). They constitute a useful dataset to evaluate the impact that current pesticide residue levels have on honey bees and, possibly, wild bees as well; this risk is different to the risk of being affected by spray drift of these plant-protection products [33], [34].

Typical risk assessments consider only acute toxicity of the chemicals either by topical or oral exposure in 24 or 48 hours, ignoring thus the negative effects derived from constant exposure to pesticide residues over longer periods. Some assessments have focused on environmental fate of pesticides and their application rates to estimate Toxicity Exposure Ratios (TERs) that were then used as indicators of the risk for honeybees due to particular exposure routes, e.g. ingestion of pollen or contact with it [35]. Recently, an individual study on pollen residues evaluates the possible risk of such residues to honey bees by both contact with and ingestion of contaminated pollen [28]. Neither study, however, includes the frequency of contaminated pollen among the risk parameters, while they also ignore the residues in honey or nectar. This we consider a serious flaw, as risk assessments should be based on the probability of exposure to actual residue levels. Indeed, none of the frequency data from the surveys mentioned above have been used to assess the impact that individual chemical residues and their combinations may or may not have on bees.

Some authors have tried to link the residue levels to the CCD in America [36], but by and large no risk assessment that includes residue levels, their prevalence and toxicity has been carried out to date. The handicap here is not insufficient residue data or acute toxicity data, but rather a lack of understanding as to how chronic toxicity by constant dietary exposure to residues found in pollen and honey affect the mortality of individual bees and the growth and reproduction of their colonies. Such effects include not only sublethal impairments but also delayed mortality [37]. Experiments with bumble bees have demonstrated that the lethal effects of new insecticidal compounds, including insect growth regulators and neonicotinoids, cannot be assessed based on acute toxicity data alone [22]. To understand the impact of small but constant doses of toxic residues on bee colonies it is necessary to apply different approaches where the time of exposure is taken into account [38].

Here, we attempt to provide a comprehensive risk assessment for all pesticide residues found in pollen and honey, or nectar, to bumble and honey bees using all residue and toxicity information available to date in the open literature and databases. Residue data originate from application of pesticides in accordance with standard agricultural practices in the countries surveyed, not from worst case, theoretical scenarios. Bees rely on nectar and pollen to meet the majority of their nutritional requirements, and therefore our risk assessment is focused on these two plant materials; honey is just concentrated nectar. Residues in wax are not included in this assessment since their availability to the bees was considered to be negligible compared to the direct exposure by contact with or dietary intake of pollen and honey [39]. However, recent research indicates that wax residues may also have an impact higher than expected until now [40], so available residue data in wax is presented for comparison only. Inhalation of volatile pesticides near treated crops is also excluded, since this is considered a minor route of exposure for most pesticides [41]. Traditional as well as novel methods of risk assessment will be used and compared in their predictions.

This assessment differs from those intended for regulatory purposes in several aspects: i) our focus is on the actual exposure of bees to the current pesticide residues found in the environment of developed and developing countries, not on the predicted exposure levels determined by models used in the tiered process of pesticide registration; ii) our assessment does not consider the particular application method of individual chemicals to their specific crops (e.g. foliar spray, granular, seed treatment, etc.) as it is based on the residue levels that are actually found in pollen and honey, regardless of the way they get there; iii) our assessment considers bee larvae and two castes of worker bees with different food requirements: nurses that feed on pollen, and nectar foragers. While the viability of the bee colonies depends largely on the queen’s health and her reproductive output, at present there is insufficient knowledge to assess the impact that pesticides have on the queen’s performance – the exception being recent studies with honey bees [42] and bumble bees [43].

The aim of this risk assessment is to identify the main chemicals that may pose a threat to the life of bees in their natural environment, which is currently contaminated with a large array of pesticides and other chemicals. By highlighting the compounds with higher risk to bees, we hope that apiculturists, beekeepers and policy makers involved in agricultural production will be able to screen the products most harmful to bees and find the appropriate remedies to avoid further damage.

## Materials and Methods

This assessment is restricted to honey bees (*Apis mellifera*) and bumble bees (*Bombus* spp.), which are very important pollinators and have been well studied. Information on ecotoxicity of a few pesticides to other wild bees exists [44], [45], [46], and their assessment can be inferred from the risk to the most common bee species presented here.

### Residues Data

Data on pesticide residues in pollen, honey and wax from bee hives were taken from several sources, including recent pesticide surveys in the USA [26], [27], France [47] and Spain [31] as well as a survey of neonicotinoids in Poland [48]. The review by Johnson et al. [17] provided further data on maximum residues in all these matrices. Residues in honey include additional data from surveys in Greece [49], Spain [50], Brazil [51] and India [52], complemented with sparse data from other sources as well as with residues in nectar from treated plants [44], [47], [53], [54], [55], [56]. Residues in wax also include other data from Spain [57] and the USA [26], [58]. The data were compiled to obtain average and maximum residue loads for each compound, and their frequency, in pollen, wax, honey or nectar (see Table S1).

### Toxicity Data

Acute oral and contact toxicity of pesticides to honey bees are available for the majority of pesticides as either median lethal doses per bee (LD50) or median lethal concentrations (LC50) in the tested media. Median values are preferred to no-observed effect level (NOEL) or the lowest-observed effect level (LOEL) values, which are only available for a small number of compounds and which relevance for risk assessment has been questioned on statistical grounds [59] and inaccuracy [60].

Toxicity data for honey bees were obtained from the Pesticide Manual [61], the ECOTOX database of the U.S. Environment Protection Agency (http://cfpub.epa.gov/ecotox/) and the AgriTox Database of the Agence Nationale de Sécurité Sanitaire de l’Alimentation, de l’Environnement et du Travail in France (http://www.agritox.anses.fr/index.php). Toxicity of 29 insecticides to bumble bees was obtained from ECOTOX and the open literature [2].

Agreement between the toxicity data sources was remarkably high (>95% of all compounds), with only a handful of compounds (8) showing obvious discrepancies. It is concerning, however, that LD50 values for 30% of the most highly toxic compounds to bees are not reported in the Pesticide Manual, since this is the database most commonly used by consultants in the agricultural business. Notable among these omissions are imidacloprid, emamectin benzoate, etofenprox, flumethrin, prallethrin and several organophosphorus compounds: dicrotophos, parathion (ethyl), omethoate and acephate. Surprisingly, toxicity data for coumaphos – which is widely used in apiaries for mite control – were absent from the Pesticide Manual and Agritox databases, as noted also by other researchers [28].

Oral toxicities were available for 221 compounds of the 322 pesticides compiled (69%), whereas contact toxicity (topical) covered 96% of the pesticides (see Table S2). Included in the data are 76% of existing insecticides and fungicides and 83% of acaricides registered for use in agriculture. Herbicides were excluded since they are non-toxic to bees, i.e. LD50 values above 100 or 200 μg bee^−1^. When more than one oral or contact LD50 value was available, a geometric mean was calculated. For data reported as “more than a given value”, that value was used in the calculations. Oral toxicities were referred in almost all cases to 48-h exposures, whereas contact exposures varied between less than a day and 96-h, with a median of 48-h, so the average LD50s or LC50s used here can be considered representative of acute exposures to bees in about 2 days.

Unfortunately, no toxicity data for larvae are available (but see [40]), so here we assume the same LD50 values for larvae as for adult bees. Chronic data for bees are extremely rare and only reported for 1 systemic insecticide [62], [63], and 6 insecticide growth regulators [22], as indicated in Table S2.

**Figure 1:**
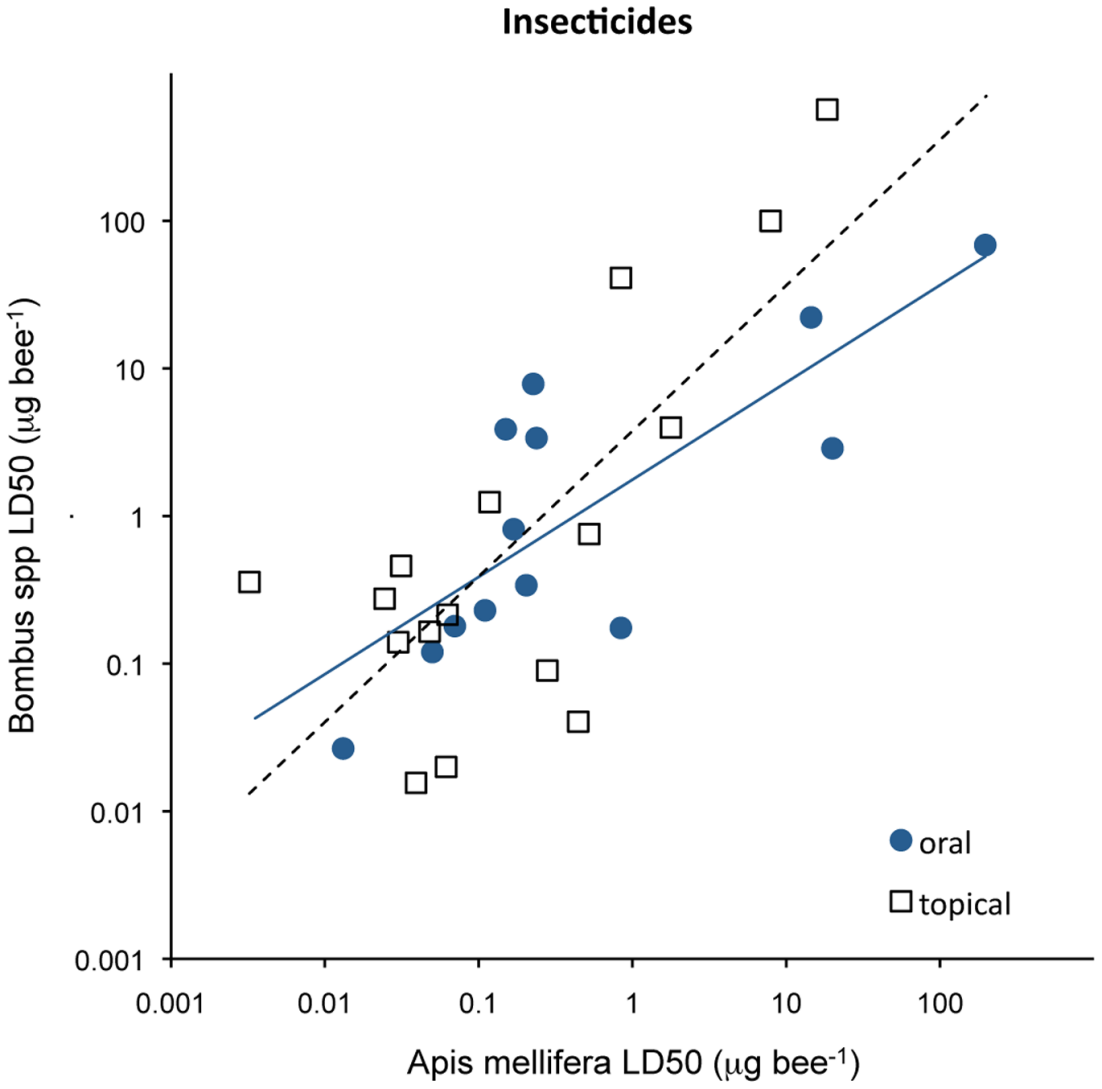
Comparison of the sensitivity of honey bees (*Apis mellifera*) and bumble bees (*Bombus* spp.) to 29 insecticides, as expressed by their contact or oral LD50s (μg bee^−1^). Susceptibility of both species by oral exposure is similar (line, slope = 0.34, p<0.001), whereas on average bumble bees are 7 times less sensitive than honey bees by contact exposure, after correcting for weight (stippled line, slope = 28.3, p<0.001).

A regression of insecticides’ LD50s (μg bee^−1^) between honey and bumble bees reveals that the sensitivity of honey bees by oral exposure is similar to that of bumble bees (slope = 0.34, r^2^ = 0.94, p<0.001, n = 13), whereas bumble bees are 28 times less sensitive than honey bees in regard to contact exposure with insecticides (slope = 28.3, r^2^ = 0.93, p<0.001, n = 16) (Fig. 1). Even after correcting for weight between species, bumble bees are about 7 times less sensitive to insecticides by contact than honey bees. Because such difference varies from chemical to chemical, extrapolations of toxicity from honey bee to bumble bee have been avoided in this study, even if they may be useful in some situations [33], [64].

### Data Analysis

#### Standard risk approach

It should be recognised that the standard hazard quotient (i.e. HQ = PEC/LD50, where PEC is the predicted environmental concentration) is not a measure of risk because it does not indicate the probability of a hazard to occur. And yet, previous studies on pesticides and bees used HQs in their evaluations [28], [35]. To estimate the risk of bees being affected by contaminated pollen or nectar it is necessary to consider the frequency of detection of pesticides residues in such matrices, because prevalence indicates the probability of exposure to the contaminants. Therefore, a simple risk assessment should incorporate this probability as follows
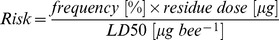
(1)


This expression indicates that a given pesticide residue has a certain probability of causing 50% mortality among the bees that come into contact with or ingest the contaminated pollen or nectar. Since we only use here LD50 values to estimate risks, our assessment should be considered very conservative. For estimation of risks at the lowest effect level, approximate estimates of LD10 can be calculated as 0.1 × LD50. Such approximation is based on previous field studies that determined the lowest effect levels of many pesticides on aquatic organisms [65].

For expression (1) to represent the actual probability of risk, residue loads should be first converted to the actual doses of residue that come in contact with (topical exposure) or are ingested (oral exposure) by the bees. Having data on average and maximum residue loads allows us establish a range of possible risks to bees. For average loads, the frequency of detection shown in Table S1 was used in the calculations; for maximum loads it should be noted that their frequency of detection is 1/total number of samples analysed in each survey. As the number of samples per survey varies between 99 and 845, the frequency of appearance of maximum residues is in the range 0.1–1.0%, i.e. much lower than the average prevalence of residues.

In the case of exposure by contact with pollen, topical LD50s shown in Table S2 were used to calculate the risk for a worker bee that comes in contact with 1 g of contaminated pollen per day. For oral exposure we focus on nurses, which feed exclusively on pollen for 10 days, and nectar foragers, which feed on nectar/honey for another 20 days during the summer season (Table 1). These types are considered representative of the bee colonies during the summer, when most pesticides are applied to crops, although winter bees can be exposed to residues in nectar for up to 100 days or more [66]. Lack of specific data on intake by bumble bees obliged us to scale the same rates as honey bees multiplied by a factor of 5, based on average intake of syrup by workers of *Bombus terrestris* and *Apis mellifera* - see File SI for estimation of contact doses and daily intake of residues.

**Table 1:** Life-span of larvae and worker bees and their consumption rates of pollen and honey (After [76]).

	Apis mellifera			Bombus spp.^1^	
	Daily rate (mg/day)		Life-span (days)	Daily rate (mg/day)	
	Honey	Pollen		Honey	Pollen
Drone larvae	15.1	1.1	6.5	75.5	5.5
Worker larvae	28.9	1.1	5	144.5	5.5
Brood attendant	34–50		8	170–250	
Nectar forager	80.2		30	401	
Nurse worker		6.5	10		32.5
Pollen forager	13		30	65	
Wax-bees	18		6	90	
Winter bees	8.8		91+	44	

1 Assuming 5 times the consumption of *Apis mellifera* in the same proportion.

#### Risk of synergistic mixtures

Because of the known synergism between ergosterol inhibiting fungicides with pyrethroids and cyano-substituted neonicotinoids (i.e. thiacloprid and acetamiprid), risks of residue mixtures were also included in this assessment for both contact and dietary exposures. These fungicides disable the monooxygenase detoxification system in honey bees, thus increasing the lethal effects several fold [67], [68], [69]. Synergistic factors vary for each combination and are reported only for topical exposure, but here we assume the same factor applies to oral exposures. In any case, the LD50 of the mixture was estimated as
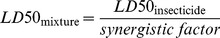
(2)


Risks of mixtures are estimated using equation (1), with residue loads of the insecticide and the combined frequency of the compounds. Since the probability of finding residues of both insecticide and synergist in the same pollen or honey cannot be estimated here we considered the lowest frequency of either compound only.

#### New approaches to risk

The above expressions of risk indicate probabilities of causing serious effects (e.g. 20% risk of resulting in 50% mortality) within short periods of exposure, i.e. about 2 days. They suit well the assessment of risks by contact exposures. However, they may not be appropriate to assess risks by chronic, dietary exposure because the bees constantly consume pollen, nectar and honey. Assuming the residues ingested remain in the body, the median lethal dose may be reached after some time; in practice, there is some elimination and metabolism for most compounds [70], so the cumulative residue amounts estimated this way represent a worse case scenario. As the residue loads in pollen and honey are already known, the only limitation is the life-span of the individual bees, which varies from 5 days in worker larvae to 100 or more days in winter worker bees (Table 1).

Consequently, a simple way to assess the dietary risk of pesticide residues is by estimating the time to reach their corresponding LD50s, and compare those times with the actual life-span of each stage of development. Only times which are shorter than the life-span would represent a serious risk, as they indicate that surely more than 50% of the bees exposed would die. We use two distinct new approaches for assessing dietary exposure:

- Fixed dose approach. Assuming that acute LD50 values are constant for each pesticide, estimates of the time to reach the dietary LD50 (henceforth T50) of each pesticide were calculated as follows
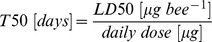
(3)As with the standard risk assessment, T50s were estimated for intake of average residues as well as maximum residues, so as to provide a range rather than an exact number of days. This approach may be valid for most pesticides, but there are some exceptions that justify another way of assessing risks.
- Time-cumulative effects. This approach is based on the experimental observation that dietary LD50s for certain compounds decrease with exposure time [37]. Consequently, the estimated T50s will be reached earlier than expected. The rate of change of LD50 with time can be estimated experimentally by a simple log-to-log regression of the LD50s on the exposure times
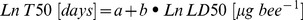
(4)where a (intercept) and b (slope) are empirical parameters specific to each chemical and species tested [71]. Slope values <1 result in an exponential increase of effects over time, according to the Druckrey-Küpfmüller equation (D Tn = constant, where the exponent n = 1/slope, D = dose and T = time) [37]. To date, there is empirical evidence of time-cumulative toxicity for some carcinogenic substances, neonicotinoid insecticides, rodenticides and methylmercury, and its underlying mechanism is thought to be the irreversible binding of the toxicant to specific receptors [72]. In the case of bees, the only data available are for the neonicotinoids imidacloprid [62], [63] and thiamethoxam [73], so this new approach will be used here only for these two compounds.

## Results

### Residue Data

A total of 161 pesticides have been found so far in bee hives, of which 124 appeared in pollen, 95 in wax and 77 in honey or nectar. The majority were insecticides (83), with fungicides (40), herbicides (27) and acaricides (10) making up the remainder; only one insecticide synergist (piperonyl butoxide) was found [17]. Among the survey’s data,15 metabolites were reported due to their toxicity and persistence (e.g. aldicarb sulfoxide, endosulfan sulfate, fipronil sulfone, 5-hydroxy-imidacloprid, DDE, etc.). Whenever metabolites are reported, total loads for a compound were calculated as the sum of loads of the individual metabolites and their respective parent compound, weighted for the frequency of appearance in the respective surveys. Some persistent compounds are no longer used in one or several of the countries surveyed (e.g. dieldrin, DDT, HCB) but their residues are still present in their environment and need to be taken into account for risk assessment of bees. Obviously not all chemicals appeared in all surveys, and their concentrations and frequency differed markedly among surveys, reflecting the usage pattern of agrochemicals in each country or region.

**Figure 2:**
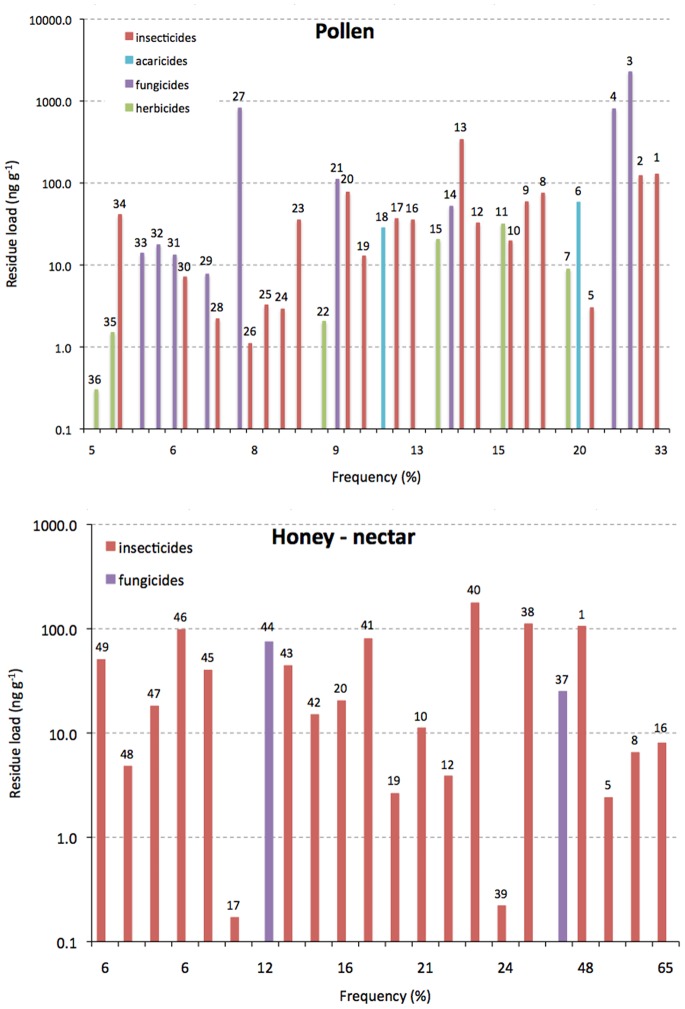
Residue loads of the most common pesticides plotted against their prevalence (frequency) in: A) pollen; B) honey or nectar. Key: 1 coumaphos (total); 2 tau-fluvalinate; 3 thymol; 4 chlorothalonil; 5 acetamiprid; 6 amitraz (total); 7 dithiopyr; 8 thiacloprid; 9 carbaryl; 10 imidacloprid (total); 11 pendimethalin; 12 chlorpyrifos; 13 phosmet; 14 carbendazim; 15 atrazine; 16 thiamethoxam; 17 chlorfenvinphos; 18 fenpyroximate; 19 clothianidin; 20 endosulfan (total); 21 thiophanate-methyl; 22 metolachlor; 23 fenpropathrin; 24 methoxyfenozide; 25 esfenvalerate; 26 tebufenozide; 27 captan (total); 28 bifenthrin; 29 azoxystrobin; 30 lambda-cyhalothrin; 31 diphenylamine; 32 penconazole; 33 trifloxystrobin; 34 fenthion; 35 norflurazon; 36 metribuzin; 37 hexachlorobenzene; 38 HCH (alpha and beta); 39 phorate; 40 gamma-HCH (lindane); 41 heptenofos; 42 methiocarb; 43 DDT (total); 44 vinclozolin; 45 methidathion; 46 malathion; 47 cypermethrin; 48 dimethoate; 49 carbofuran (total).

The highest residue concentrations were found in wax and pollen (average 126 and 66 μg kg^−1^ respectively), whereas the highest frequency of detection corresponds to wax (over 50% for chlorfenvinphos, tau-fluvalinate, bromopropylate, coumaphos and chlorothalonil) and honey (over 50% for thiacloprid, thiamethoxam and acetamiprid [48]). It is unclear whether the residues detected in pollen collected from apiaries (Fig. 2a) originated from sprayed fields or from hives treated with pesticides; they are probably a mixture of both. Whatever their source, such pollen feeds the nurse bees and larvae. Among the residues in honey, systemic insecticides stand out for their high prevalence: neonicotinoids are the most commonly found, while phorate, dimethoate and carbofuran are typically present in more than 5% of nectar collected from treated plants (Fig. 2b). The presence of hydrophobic pesticides such as coumaphos or vinclozolin, and to a lesser extent tau-fluvalinate, in honey suggests contamination from the comb, since honey bee colonies are commonly treated with these pesticides for mite and fungal control [74].

### Risk by Contact Exposure

A total of 92 individual compounds could be assessed for risk to contaminated pollen by contact exposure after matching residue and toxicity data. To these were added the synergistic combinations of cyhalothrin, thiacloprid and acetamiprid with three ergosterol inhibiting fungicides: propiconazole, penconazole and myclobutanil. Table 2 shows the risk for honey bees and bumble bees exposed to average and maximum residue levels of each compound, after taking into account their average prevalence in Europe, America and Asia. Only 33 compounds and 5 mixtures that have some relevance (i.e. risk >0.1) are shown, since 65% of the compounds have negligible or no risk to the bees. Risks above 5% can be considered high, as they correspond to T50s of 2 days or less; between 1 and 5% the risk is moderate, usually corresponding to T50s between 2 and 7 days, which are within the life-span of larvae and adult workers; risk below 1% can be regarded as low, for which T50s range from 7 to 60 days and more, covering the life-span of nectar foragers in summer and most of the life-span of winter bees.

**Table 2:** Risk (% probability) and time to reach topical LD50 (T50 in days) for worker bees, estimated as contact exposure with 1 g of contaminated pollen, at average or maximum levels, during 2 days.

Use^1^	Chemical	Honey bee					Bumble bee				
		Topical LD50 (μg bee^−1^)	Risk (%)		TD50 (days)		Topical LD50 (μg bee^−1^)	Risk (%)		TD50 (days)	
			Average	Max	Average	Max		Average	Max	Average	Max
I	thiamethoxam	0.02	29.58	3.66	1	0.2					
I	phosmet	0.62	14.56	23.89	2	0.04					
I	chlorpyrifos	0.07	12.92	10.33	2	0.1	0.09	10.32	8.26	3	0.1
I	imidacloprid (total)	0.06	10.34	16.00	3	0.1	0.02	31.77	49.15	1	0.02
I+F	cyhalothrin+propiconazole	0.003^2^	8.79	5.90	0.4	0.1	0.01^2^	2.56	1.71	1.4	0.3
I+F	cyhalothrin+myclobutanil	0.004^2^	7.86	5.52	0.6	0.1	0.02^2^	2.28	1.60	2.1	0.4
I+F	cyhalothrin+penconazole	0.011^2^	7.23	3.68	2	0.3	0.04^2^	2.10	1.07	5	1.0
I	clothianidin	0.04	5.28	0.99	4	1	0.02	13.26	2.49	2	0.4
I+F	thiacloprid+propiconazole	0.065^2^	4.21	7.43	1	0.1					
A	acrinathrin (total)	0.17	3.44	1.21	1	0.2					
I	deltamethrin	0.02	3.27	2.26	1	0.3	0.28	0.29	0.20	11	3
I-A	cypermethrin	0.03	2.40	1.75	2	1					
I	carbaryl	0.84	2.32	0.95	14	1	41.16	0.05	0.02	699	39
I	bifenthrin	0.01	1.97	1.14	7	1					
I	esfenvalerate	0.03	1.96	2.99	8	0.4					
I	fenthion	0.22	1.89	0.57	5	1					
I	dinotefuran	0.05	1.84	2.18	1	0.3					
I	lambda-cyhalothrin	0.05	1.83	0.71	7	1	0.17	0.53	0.21	23	5
I	fipronil (total)	0.007	1.19	2.49	5	0.3					
A	pyridaben	0.05	1.00	0.29	3	2					
I-A	tau-fluvalinate	8.66	0.92	0.28	70	3					
I	indoxacarb	0.59	0.81	0.43	5	1					
I	permethrin	0.06	0.60	1.90	6	1	0.22	0.18	0.55	20	2
I	beta-cyfluthrin	0.03	0.56	1.41	14	1	0.46	0.04	0.10	209	14
I	prallethrin	0.03	0.42	0.16	4	4					
I-A	coumaphos (total)	20.29	0.41	0.28	158	3					
I	phenothrin	0.13	0.38	0.37	2	2					
F	chlorothalonil	135.32	0.32	0.35	169	1					
I-A	endosulfan (total)	6.35	0.26	0.48	82	2					
I-A	carbofuran (total)	0.16	0.22	0.55	13	1					
I-A	chlorfenvinphos	4.10	0.22	0.05	112	10					
I-A	aldicarb (total)	0.38	0.17	1.61	29	0.3					
I-A	methomyl	0.49	0.16	0.03	48	21					
I+F	acetamiprid+propiconazole	0.076^2^	0.14	0.85	25	0.6	0.95^2^	0.01	0.07	318	7.1
I-A	diazinon	0.38	0.14	0.05	45	9					
I	acephate	1.78	0.11	0.06	20	11	3.99	0.05	0.02	44	24
I-A	malathion	0.47	0.11	0.07	28	7					
I+F	acetamiprid+fenbuconazole	1.76^2^	0.01	0.09	587	13	22.2^2^	0.00	0.01	7407	166
IGR	diflubenzuron	114.80	0.00	0.00	1441	897	0.10^3^	1.68	1.66	1	1

1 A = acaricide; F = fungicide; I = insecticide; IGR = insect growth regulator; IS = insecticide synergist.

2 Mixture LD50 estimated in accordance with known synergistic ratios [67], [68], [69].

3 Chronic LD50 for 77 days exposure.

Not surprisingly, the bulk of chemicals posing contact risk to bees are insecticides (20) or insecticide-acaricides (10), with only 2 acaricides, 1 fungicide and 5 fungicide mixtures appearing in that list. The risk of being seriously affected by contact with pollen residues is generally low, with only 5 compounds showing high risks (>5%): thiamethoxam (3.7–29.6% for honey bees), phosmet (14.6–23.9% for honey bees), chlorpyrifos (8.3–12.9% for both bees), imidacloprid (10.3–16% for honey bees but 31.8–49% for bumble bees) and clothianidin (1.0–5.3% for honey bees and 2.5–13.3% for bumble bees). It should be noted that the risk of these neonicotinoids to bumble bees is about two to three times as high as for honey bees, due to the different sensitivity among the species (Fig. 1). These compounds pose high risk to bees on account of their extremely high toxicity to both honey and bumble bees, with topical LD50s in the range 0.02–0.09 μg bee^−1^, and also because their average residues (12–35 ppb) were present in 11 to 16% of the pollen surveys worldwide. By contrast, the high risk of phosmet is mainly due to average residues of 339 ppb (highest 16.5 ppm) in spite of its moderate toxicity to honey bees (topical LD50 = 0.62 μg bee^−1^). While six other compounds were more common among the residues (coumaphos, tau-fluvalinate, chlorothalonil, acetamiprid, amitraz and thiacloprid), their toxicity to bees is 100–5000 times lower than that of thiamethoxam or chlorpyrifos.

Mixtures of fungicides with cyhalothrin or thiacloprid pose also high risks for honey bees (3.7–8.8%) and a moderate risk to bumble bees (1.1–2.6%), even if the prevalence of the three fungicides is relatively low (1.8–5.5%). Attention should be paid to the synergism of propiconazole with such insecticides, as it changes markedly the risk of the individual compounds from being moderate (0.2–1.8% cyhalothrin) or negligible (<0.1% thiacloprid) to a high risk. The synergistic factor of propiconazole for thiacloprid is 560 [67], [75] and for cyhalothrin 16.2 [67], [68]. Only the mixtures acetamiprid with propiconazole and fenuconazole showed low risk for honey bees (0.1–0.7%) and negligible risk for bumble bees (0.01–0.07%) based on synergistic factors of 100-fold (propiconazole) or 4.5-fold (fenuconazole) and the low frequency of such fungicides in pollen (1.8–3.3%).

Moderate risk by contact exposure (i.e.1–5%) includes 6 pyrethroids (acrinathrin, deltamethrin, cypermethrin, bifenthrin, esfenvalerate and lambda-cyhalothrin, in that order), the carbamate carbaryl, the organophosphorus fenthion, the neonicotinoid dinetofuran, the pyrazole fipronil and the acaricide pyridaben. However, risks of these compounds to bumble bees are below 1%, because they are less toxic to the large pollinators (Table S2). Notice that, despite fipronil and bifenthrin being among the most toxic insecticides to honey bees (topical LD50 0.007 and 0.015 μg bee^−1^ respectively), their risk by contact exposure is reduced because of their low residue loads (1.6–29 ppb and 2.2–13 ppb respectively) and low prevalence in pollen residues (average 2.8 and 6.6% respectively).

The remaining 17 compounds pose a low risk (<1%) for being less toxic (chlorothalonil, coumaphos, tau-fluvalinate, endosulfan, chlorfenvinphos), appearing rarely (phenothrin, prallethrin, acephate, carbofuran, malathion and permethrin) or both (diazinon, methonyl, aldicarb, beta-cyfluthrin and indoxacarb). Except for pyrethroid residues, which can have almost immediate effects by contact exposure, the average T50 for all other pesticides in this group is above 60 days for honey bees, denoting a very low risk by contact with pollen. Obviously, maximum residues of these pesticides would result in serious effects in very few days or even less (Table 2). Presumably, a similar risk would apply to contact with residues in wax.

### Risk by Dietary Exposure

Information on oral toxicity to bees is less comprehensive than that of topical toxicity (see Table S2), so only 77 compounds could be evaluated here. Average and maximum daily doses of residues ingested (Table S3) were calculated first to assess the risk of worker larvae, nurses and nectar foragers when exposed to the array of pesticides found in pollen and nectar (Table S1). Considering the life spans of each type of bee, the risk of consuming contaminated food during their lifetime and the T50 were assessed using the standard risk method. Results for 25 pesticides and 1 mixture that pose some risks (i.e. >0.1%) to honey bees are shown in Table 3; the remaining 67% of pesticides pose a negligible or no dietary risks to these bees.

**Table 3:** Risk (% probability) and time to reach oral LD50 (T50 in days) for larvae and workers of honey bees feeding on contaminated pollen and/or nectar at average or maximum residue levels (see Table S3).

Chemical	Oral LD50 (μg bee^−1^)	Risk (%)						T50 (days)					
		Worker larvae^1^		Nurses^2^		Nectar forager^3^		Worker larvae		Nurses		Nectar forager	
		Average	Max	Average	Max	Average	Max	Average	Max	Average	Max	Average	Max
thiamethoxam	0.005	2.77	0.23	4.80	0.59	200.18	3.86	23	8	27	6	10	4
gamma-HCH (lindane)	0.05	0.62	4.02	0.01	0.01	200.40	313.09	9	0.4	979	323	3	0.1
imidacloprid (total)	0.013	1.19	0.64	1.57	2.43	23.33	5.93	68	4	103	2	28	2
clothianidin	0.004	1.02	0.23	1.91	0.36	22.04	3.25	54	10	58	13	23	4
cypermethrin	0.06	0.13	0.10	0.04	0.03	4.00	6.77	119	23	711	154	44	9
coumaphos (total)	4.61	0.11	0.03	0.06	0.04	2.62	4.37	1444	71	5524	120	545	28
dinotefuran	0.02	0.10	0.06	0.13	0.16	1.50	2.37	49	27	74	20	20	13
quinalphos	0.07	0.00	0.00			1.29	0.69	253	236			91	85
methiocarb	0.47	0.00	0.00	0.00	0.00	1.08	0.28	1080	601	51648	51648	391	217
chlorpyrifos	0.24	0.04	0.01	0.13	0.10	0.86	0.30	1605	176	1118	44	764	197
carbaryl	0.15	0.41	0.03	0.42	0.17	0.54	0.82	202	63	392	22	80	45
beta-cyfluthrin	0.05	0.10	0.03	0.01	0.03	0.43	0.62	190	123	3497	226	69	49
dimethoate	0.17	0.01	0.00	0.00	0.00	0.40	0.24	1198	662	11303	6190	440	243
DDT (total)	5.08	0.00	0.00	0.00	0.00	0.29	0.62	3871	266	25061	7108	1432	96
pirimiphos ethyl	0.22	0.00	0.00			0.21	0.24	401	346			144	125
diazinon	0.21	0.04	0.01	0.01	0.00	0.19	0.39	426	202	3869	781	156	76
malathion	9.17	0.00	0.00	0.00	0.00	0.15	0.12	3218	1292	82696	20162	1167	471
pirimicarb	3.84	0.00	0.00			0.10	0.09	3500	1873			1261	675
thiacloprid+propiconazole	0.03^4^	0.08	0.29	0.30	0.53	0.00	0.00	109	4	61	5	57	2
phosmet	0.37	0.07	0.11	0.79	1.29			991	20	168	3		
fipronil (total)	0.001	0.02	0.05	0.27	0.57			596	33	101	6		
acrinathrin (total)	0.12	0.01	0.36	0.17	0.06		49.74	719	2	122	20		1
acephate	0.23	0.00	0.01	0.03	0.01		0.55	2264	135	383	214		54
permethrin	0.13	0.00	0.02	0.01	0.03		0.52	10842	142	1835	209		58
methoxychlor	5.02	0.00	0.00				0.28		293				106
dichlorvos	0.29	0.00	0.00	0.00	0.00	0.07	0.11	1215	735	6561	4746	452	272

1 Exposure period of 5 days.

2 Exposure period of 10 days.

3 Exposure period of 30 days.

4 Mixture LD50 estimated in accordance with known synergistic ratios [67].

#### Dietary risk to honey bees

Extremely high risks were found for thiamethoxam and lindane residues in honey, which affect primarily nectar foragers and secondarily the larvae. Daily consumption of nectar or honey contaminated with these compounds at the average residue levels found worldwide would cause nectar forager mortalities of 50% or above within 3 days in the case of lindane, or a week for thiamethoxam (Table 3). The risk of these two insecticides to larvae is moderate (0.6–4.0% lindane, 0.2–2.8% thiamethoxam), since larvae consume less amounts and their exposure is only during 5 days. In addition, two other neonicotinoid insecticides found in honey pose high risks to foragers (3–22% clothianidin, 6–23% imidacloprid) and moderate risks to larvae (0.2–1.2% for either compound).

Residues of the pyrethroid cypermethrin in honey pose a moderate risk to nectar foragers (4.0–6.8%) but a low risk to larvae (0.1%). Moderate risks (1–5%) are also found for the organophosphorus coumaphos and quinalphos, the neonicotinoid dinetofuran and the carbamate methiocarb, but only coumaphos and dinetofuran present some risk to larvae. Nectar foragers are at low risk (0.1–1%) when feeding on honey contaminated with 9 more insecticides: the organophosphorus chlorpyrifos, dimethoate, pirimiphos ethyl, diazinon and malathion, the carbamates carbaryl and pirimicarb, the pyrethroid beta-cyfluthrin and total residues of DDT (i.e. DDT and its metabolites). Among these, only carbaryl seems to pose a minor risk to bee larvae (0.03–0.4%) and foragers (0.5–0.8%) alike, but daily consumption of its residues would only inflict some mortality among the adult foragers (T50 of 45–80 days). Residues of the synergistic fungicides, myclobutanil, penconazole and propiconazole have so far not been detected in honey, and therefore nectar foragers are exempt of higher risks in this regard.

The residual composition of pollen is different from that of honey, with 70 out of the 124 pesticides found only in pollen (Table S1). Among the worker bees, only nurses depend entirely on this kind of food, but the queen and larvae are fed substantial amounts of pollen as well ([76], Table 1). Moderate risks of pollen residues (1–5%) to both nurses and larvae were found for thiamethoxam, clothianidin, imidacloprid and phosmet. Estimated T50s for thiomethoxam are 6–27 days for nurses and 8–23 days for larvae, depending on the residue load. Obviously, the high toxicity of this insecticide to honey bees (oral LD50 0.005 μg bee^−1^), together with its relatively high residue loads (29 ppb) and worldwide prevalence (12.8%) are the main reasons behind this risk. Although clothianidin is 4 times more toxic than imidacloprid, average residues of the latter insecticide are slightly higher and more frequently found in pollen than those of the former, so their overall risk is very similar. Nevertheless, only the highest residues of imidacloprid would seriously affect nurses and larvae alike, with T50s of 2 and 4 days respectively, whereas the highest clothianidin residues would have a smaller impact because the T50s are longer than the life spans of the bees (Table 3). In addition to neonicotinoids, the highest residues of phosmet and fipronil in pollen can result in 50% mortality of nurses in 3 and 6 days, respectively; but the risk can be considered low due to their low average residues (0.8% phosmet and 0.3% fipronil).

Residues in pollen of 4 other insecticides (carbaryl, acrinathrin, dinotefuran and chlorpyrifos) have low risk to honey bees (0.1–1%), as their T50s exceed by a long margin the life spans of nurses and larvae (Table 3). Also, the mixture of thiacloprid+propiconazole may pose some risk to larvae and nurses (T50s of 4 and 5 days respectively) only when residues of thiacloprid in pollen are at the highest recorded levels (1 ppm); otherwise, under normal circumstances the average residues of this neonicotiniod in pollen (75 ppb) wouldn’t be of concern to either forager bees (0.5%, and T50 57 days) or larvae (0.08%, and T50 109 days). Risk of the remaining compounds found in pollen is considered negligible.

#### Dietary risk to bumble bees

In the case of bumble bees, estimates of risks for 15 compounds and 4 mixtures for which toxicity data are available are shown in Table 4. Contrasting with honey bees, the dietary risk of imidacloprid to bumble bees is very high: 14.5–57.4% for nectar foragers that consume honey or nectar and 3.8–6% for nurses that feed on pollen, while a moderate risk (1.6–2.9%) was found for worker larvae that consume both types of food (Table 4). Moreover, the maximum imidacloprid residues ingested by the different types of bumble bee would reach the oral LD50 within their respective life spans, while average residues in honey result in T50 of 11 days for nectar foragers, indicating than half of them would probably die before reaching the end of their lives. In addition, residues of heptenophos in honey present high risk to forager bumble bees (10.4–29% and T50 6–17 days) but not to their larvae. Lack of toxicity data for thiamethoxam on bumble bees prevents us from a further assessment of this toxic compound using standard methods, even if some researchers have proven its negative effects on experimental bee colonies [42], [43].

**Table 4:** Risk (% probability) and time to reach oral LD50 (T50 in days) for larvae and workers of bumble bees feeding on contaminated pollen and/or nectar at average or maximum residue levels (see Table S3).

Chemical	Oral LD50 (μg bee^−1^)	Risk (%)						T50 (days)					
		Worker larvae^1^		Nurses^2^		Nectar forager^3^		Worker larvae		Nurses		Nectar forager	
		Average	Max	Average	Max	Average	Max	Average	Max	Average	Max	Average	Max
imidacloprid (total)	0.03	2.93	1.57	3.86	5.98	57.39	14.58	28	2	42	1	11	1
heptenophos	0.53	0.00	0.00			28.98	10.42	46	16			17	6
chlorpyrifos	0.23	0.23	0.07	0.65	0.52	4.44	1.57	313	34	218	9	149	38
quinalphos	0.18	0.00	0.00			2.51	1.34	130	122			47	44
beta-cyfluthrin	0.12	0.22	0.05	0.02	0.06	0.90	1.28	92	59	1683	109	33	23
dimethoate	0.82	0.01	0.00	0.00	0.00	0.41	0.25	1159	641	10930	5986	425	235
lambda-cyhalothrin	0.18	0.02	0.00	0.08	0.03	0.19	0.10	1248	567	757	149	624	572
carbaryl	3.88	0.08	0.01	0.08	0.03	0.10	0.16	1050	328	2034	114	415	231
cyhalothrin+propiconazole	0.01^4^	0.12	0.03	0.11	0.13	0.19	0.10	77	35	47	9	624	572
cyhalothrin+myclobutanil	0.02^4^	0.11	0.03	0.07	0.12	0.19	0.10	114	52	69	14	624	572
cyhalothrin+penconazole	0.04^4^	0.10	0.02	0.03	0.08	0.19	0.10	282	128	171	34	624	572
acetamiprid+propiconazole	0.21^4^	0.02	0.02	0.00	0.02	0.07	0.00	585	80	2179	49	23125	4173
acetamiprid	22.20	0.00	0.00	0.00	0.00	0.07	0.00	61259	8370	228262	5110	23125	4173
diflubenzuron	1.46	0.00	0.00	0.02	0.02			3349	2084	567	353		
bifenthrin	0.34	0.00	0.00	0.01	0.01		0.11	28186	675	4770	807		284
rotenone	0.83	0.00	0.00	0.00	0.00			6538	6538	1106	1106		
acephate	7.87	0.00	0.00	0.00	0.00		0.08	15762	937	2667	1488		378
phosalone	3.98	0.00	0.00	0.00	0.00			23177	23177	3922	3922		
methomyl	3.38	0.00	0.00	0.00	0.00			59814	25670	10122	4344		

1 Exposure period of 5 days.

2 Exposure period of 10 days.

3 Exposure period of 30 days.

4 Mixture LD50 estimated in accordance with known synergistic ratios for honeybees [67], [68].

Moderate risks to nectar foragers (1–5%) were determined for the organophosphorus chlorpyrifos and quinalphos as well as the pyrethroid beta-cyfluthrin, but once again the risk of theses insecticides to larvae are low or negligible (Table 4). Based on the estimated T50s, only the highest residues of chlorpyrifos in pollen and beta-cyfluthrin in honey may represent a considerable risk to nurses and foragers respectively.

Residues of dimethoate, lambda-cyhalothrin, bifenthrin and carbaryl in honey pose a low risk to forager bumble bees. The risk of fungicides mixtures with cyhalothrin and acetamiprid is estimated as low as well. Since the three fungicides are only present in pollen, their synergism only affects the larvae and nurses, and even then the resulting risks are low: <0.12 for larvae and <0.13% for nurses, with T50s above their life span (Table 4). The only exception is with the highest residues of cyhalothrin in pollen (36 ppb), for which a T50 of 9 days was estimated.

### Risks by Cumulative Toxicity

Estimates of T50s for imidacloprid and thiamethoxam in honey bees were also carried out using the time-cumulative approach (Table 5). Indeed, the time to reach their oral LD50 is shorter than the T50s estimated by the standard method. For example, nurse bees feeding on pollen contaminated with imidacloprid would reach their LD50 in 7 to 9 days (within their life-span), and those ingesting thiamethoxam would die in large numbers after one day, no matter what the residue loads are. The same applies to nectar foragers, which would be at serious risk when feeding on nectar or honey contaminated with either chemical, and so would be the larvae consuming thiamethoxam.

**Table 5:** Comparison of estimated times to LD50 (T50 range in days) for dietary exposure of honey bees to two neonicotinoid insecticides, using standard and cumulative risk approaches.

	Imidacloprid			Thiamethoxam		
Risk approach	T50 (days)			T50 (days)		
	Worker larvae	Nurse	Nectar forager	Worker larvae	Nurse	Nectar forager
Cumulative	6–10	7–9	4–8	<0.1–1	0.2–1	<0.1–0.5
Standard	4–68	2–103	2–28	8–23	6–27	4–10
	Experimental data^1^			Experimental data^2^		
Exposure (days)	2	4	31	1	5.2	8.04
Oral LD50 (ng bee^−1^)	28.5	10.79	0.18	0.109	0.057	0.009
Equation	Ln T50 = 2.55−0.53 *Ln LD50			Ln T50 = −1.03−0.70 *Ln LD50		
Power exponent(n = 1/slope)	1.89			1.44		
r2	1.00			0.68		

1 Sources: [62], [63].

2 Source : [73].

This approach should apply to all insecticides that exhibit time-cumulative toxicity, which requires the binding to specific receptors to be persistent [72]. However, it would not apply to the majority of chemical residues found in pollen or nectar. Although fipronil and its toxic metabolites have systemic properties and high toxicity to bees [77], so far there is no evidence of time-cumulative effects of fipronil on bees or other organisms. Also, residues of pyrethroid insecticides have little effect when ingested by bees, as they are either metabolised or quickly eliminated; consequently, their oral toxicity is on average 3 times lower than their topical toxicity (Table S2). Organophosphorus and carbamate compounds undergo a similar fate, so only a handful of persistent compounds (i.e chlorpyrifos, coumaphos and chlorfenvinphos) may last long enough to cause time-dependent toxicity, if any, during chronic ingestion of their residues.

## Discussion

Bees can be exposed to plant protection products in two ways:

- by direct exposure to either drift droplets, which are scattered during the foliar spraying of crops [33], dust from seed drilling at planting [78], or inhalation of volatile pesticides during or after application to the crops; and
- by exposure to residues present in pollen, wax, nectar, honey and guttation drops, which may result either from direct spray contamination of flowers, translocation through the treated plants or soil [20], [54], or direct contamination during treatment of the combs (for honey bees only). Bees also drink water [79], and we have observed them drinking from paddy field waters contaminated with pesticides.

Our risk assessment in this paper deals only with residues in pollen and honey or nectar, because these constitute the essential food of bees [39]. Exposure to residues in these matrices may be by contact, while gathering pollen in the field and in the storages of the comb, or most likely by dietary and chronic ingestion of contaminated nectar, honey and pollen. Foragers presumably feed on nectar from flowers, rather than consuming the honey stores. Nectar is carried in the insect’s honey stomach, and then processed by the bees before it becomes honey. Foragers carry and process far more nectar than they consume. We do not know how much of the active ingredients enter the insects during these processes, but it can be assumed that the exposure of foragers could thus be much higher than estimated here. Exposure to guttation drops was not considered here, as it is unlikely to affect most bees since such drops only appear in the early hours of the day [80]. For risk during agricultural operations the reader can consult [81], [82].

The large number of agricultural chemicals found in pollen demands a rigorous evaluation of their risk to bee pollinators. Of the 124 parent compounds found in pollen from honey bee apiaries, about half of them appear with a frequency of 2% or more, 20 are present in more than 10%, and two insecticide-acaricides (coumaphos and tau-fluvalinate) appear regularly in more than 30%, particularly in North America [26], [27]. It is also worrying that residues of the four most common compounds (tau-fluvalinate, coumpahos, thymol and chlorothalonil) are present at average concentrations above 100 ng g^−1^ of pollen (Fig. 2a). Highest residues can be up to 20 times higher (see Table S1), although they only appear occasionally.

Some 77 compounds have also been found in honey, with 23 of them being exclusive to this matrix. Residues in honey include mainly systemic compounds, among which the most commonly found are neonicotinoid insecticides – up to 65% prevalence (Fig. 2b). Systemic insecticides can move from the soil, where they are applied as granules or seed-coatings, through the sap of the plants and reach the nectar glands at the time of pollination, when the bees are attracted to the flowers [83]. It is no surprise, therefore, that many residues found in honey are of hydrophilic herbicides (5) and fungicides (15), as they are known to translocate within the various parts of the treated plants [84], [85]. The highest residue loads in honey, however, correspond to hydrophobic compounds such as lindane and coumaphos, the latter being used to treat the combs for mite control [86].

Traditional risk assessments have considered only the residue loads in pollen and the acute oral or contact toxicity of the compounds [28], [33]. We draw attention here to this important distinction, as the toxicity of hydrophobic insecticides and acaricides is mostly by contact exposure whereas the toxicity of hydrophilic fungicides and systemic insecticides is mainly by oral ingestion of residues in pollen and honey. It should be noted that pyrethroids, which are highly hydrophobic compounds, are on average 3 times more toxic to bees by contact than by oral exposure. By contrast, 60% of the systemic (hydrophilic) pesticides have oral toxicities higher than their contact toxicities, up to 11 and 13 times higher in the case of clothianidin and phorate, respectively (Table S2). It follows that regulators should pay more attention to dietary toxicities of any hydrophilic compound suspected of getting into the food chain.

Also essential to any risk assessment are not just the actual residue loads, but the frequency with which they appear in pollen and/or nectar. This is because the risk of bees being affected by pesticide residues is directly proportional to the prevalence of such residues in the environment (see equation (1)). For example, chlorpyrifos and methomyl have equal oral toxicities to honey bees (0.24 ng bee^−1^), so assuming equal residue loads in pollen their hazard quotient is the same. However, because chlorpyrifos is present in 14.3% of pollen and methomyl only in 3.8%, a nurse bee is more likely to be intoxicated with the former compound while feeding on pollen, and hence the risk of chlorpyrifos to the bees is greater than that of methomyl.

When considering the relative weight of these three factors, residue loads, prevalence and toxicity, in the estimation of risks, it is evident that toxicity is the most important factor. Thus, risks above 1% by contact exposure are obtained for compounds with topical LD50s of 1 μg bee^−1^ or below, and this includes fipronil, four neonicotinoids (clothianidin, dinetofuran, imidacloprid and thiamethoxam), all synthetic pyrethroid insecticides (except tau-fluvalinate), pyridaben and six cholinesterase inhibitors: phosmet, chlorpyrifos, carbaryl, carbofuran, fenthion and aldicarb (Table 2). Risks by contact with residues in pollen or wax are likely overestimated here because not all residues are bioavailable by this exposure route [39]. High acute toxicity also determines the risk through dietary exposure, with neonicotinoids, cypermethrin, lindane and three cholinesterase inhibitors (coumaphos, quinalphos and methiocarb) posing the highest risks for honey bees (Table 3). In stark contrast, low risks were determined for three acaricides used in apiaries to control mites, tau-fluvalinate (0.3–1%), coumaphos (0.3–0.4%) and chlorfenvinphos (0.05–0.2%), even if their residues loads (36–128 ppb) are above the 60 ppb average and appear in pollen with a frequency of 12 to 32% ([26], Table S1). All of them present little risk to the bees because their toxicities by contact are low (4, 8 and 20 ng bee^−1^). They may be of concern, however, when present in high concentrations, and it is only then that they can reach the topical LD50 in 2 to 4 days (Table 2).

The second factor used in the estimation of risk is a combination of both residue loads and prevalence. Indeed, the risk of 10 ppb of residues of a compound appearing in 10% of the pollen is equivalent to the risk of 100 ppb of the same compound appearing only in 1% of pollen. For example, among the highest average residue loads found in pollen are the fungicides captan (821 ppb) and chlorothalonil (802 ppb); chlorothalonil is of greater concern (risk 0.32%) not only because is twice as toxic as captan (135 μg bee^−1^ vs 215 μg bee^−1^) but also its residues are found in 27% of the pollen, whereas captan is present only in 7% of the pollen.

Since toxicity is the main factor affecting risk, the synergistic combinations of ergosterol inhibiting fungicides with pyrethroids and cyano-substituted neonicotinoids are of great concern for one reason: the intrinsic toxicity of the individual compounds is already very high in the case of pyrethroids, and it is boosted up to 16-fold when propiconazole is present among the residues [68]. For thiacloprid the synergistic factor is as high as 560-fold, and for acetamiprid 100-fold [67], so their safety features [87] become all of a sudden hazardous. However, combinations of anilinopyrimidine fungicides with the same neonicotinoids do not show synergism in bees [75], perhaps because they do not interfere with the P-450 detoxification system. The relatively low prevalence of these fungicides among pollen residues (1.8–5.5%) could be an ameliorating factor for nurses, queens and larvae, while nectar foragers would not be affected by the synergism as honey appears to be free of these fungicides. Not considered here is the synergism of chlorothalonil with fluvalinate and coumpahos (which only occurs at high concentrations of fluvalinate), because the presence of coumaphos significantly reduces the toxicity of the fluvalinate and chlorothalonil mixture [40]. The risks of fungicide-insecticide mixtures calculated here are based on the lowest prevalence among the fungicide-insecticide pairs, but even then they may be overestimated: it is obvious that not all pollen contaminated with the insecticides (e.g. 6.2% lambda-cyhalothrin and 17.7% thiacloprid) contains at the same time residues of the synergistic fungicides. Also, risks of some residue mixtures are high for contact exposures (Table 2), but low or negligible for dietary exposures (Tables 3 and 4). Experimental evidence has shown that mixtures of imidacloprid and lambda-cyhalothrin increase mortality of bumble bees (*B. terrestris*) and reduce brood production in their colonies more than when fed on pollen contaminated with only one insecticide [88]. However, the effects of insecticide mixtures are additive, not synergistic.

The risk of dietary exposure was estimated for representatives of three different types of bees (larvae, nurses and nectar foragers) in the assumption that ingested residues and/or toxic metabolites [89] remain in their bodies. As mentioned above, this is a worst-case scenario, since elimination and metabolism over time are not taken into account; therefore, dietary risks may be overestimated for some compounds in this assessment. Although only a handful of individual pesticides appear to pose a serious threat to the bees (Tables 3 and 4), we should not forget that our evaluation considered oral LD50 values, not NOEL or LOEL values. For example, nurse honey bees feeding on pollen contaminated with imidacloprid may never reach the oral LD50 for that insecticide during their short life-time of 10 days, but toxic effects will be felt among those bees well before reaching the median dose, including some mortality. There is ample evidence that honey bees and bumble bees exposed to relevant sublethal doses of imidacloprid suffer motor and learning difficulties [90], [91], [92] and may even die in small proportions [88]. More meaningful risk assessments can be done using 1/10 of the oral LD50 values, which represent the lowest doses required for causing toxic effects. In this way, the probabilities of risk shown in Tables 3 and 4 would increase 10 times, and the T50s will be reduced correspondingly.

What is clear from the dietary assessment shown here is that systemic insecticides rank at the top of the list of risky chemicals: thiamethoxam, clothianidin, imidacloprid, dinetofuran, and to a lesser extent methiocarb, dimethoate and carbaryl [93]. These are more likely than any other pesticide to produce long-term toxic effects in workers and larvae of bumble and honey bees. However, while the systemic aldicarb is known to translocate to nectar and affect bees in the first four weeks after treatment of plants [94] its residues have not been found in honey from apiaries in recent years. In view of these findings, banning of some neonicotinoids by the European Community seems to be justified alone on the grounds of residues in the food of bees, apart from other considerations [20] and side-effects that these compounds may have [95]. Surely, the high prevalence of neonicotinoids in honey (17–65%) is of great concern not only for worker bees but also for larvae (Fig. 2b and Table 3). Presumably, queens would be affected in a similar way as larvae, because both consume royal jelly and pollen, with the queens consuming larger quantities. Some experimental evidence indicates that the reproductive output of bumble bee queens is seriously curtailed when fed on pollen contaminated with imidacloprid [88] or thiamethoxam [43].

Moreover, the risk of neonicotinoids by dietary exposure above appears to be underestimated because it is known that these insecticides have chronic toxicities that exceed the known acute toxicities [62], [73], [96]. Time-cumulative effects justify a new approach to calculate T50s based on the exponential effects with time during dietary exposure. Indeed, mortality of bees increases by a power factor of 1.5 to 2, so the LD50s are reached sooner than expected. Consequently, average residues of thiamethoxam found in honey and pollen would approach the oral LD50 within the life span of larvae and worker honey bees, while average residues of imidacloprid would cause more than 50% mortality among nectar foragers and nurses and substantial mortality among larvae (Table 5). The latter predictions are deemed more realistic than the standard risks as they are in agreement with the negative effects of these insecticides observed in laboratory and semi-field experiments [63], [97]. They contrast, however, with the conclusions of a recent report, funded by the chemical industry, suggesting that residues of thiamethoxam in pollen and nectar of oilseed rape and maize do not reduce the performance of honey bee colonies [98]. It should also be noted that sublethal and side-effects of neonicotinoids, such as immune suppression [99], have not been taken into account in our assessment.

Among the hydrophobic pesticides, the highest risks by dietary exposure correspond to four organophosphorus compounds (coumaphos, chlorpyrifos, heptenophos and quinalphos) on account of their high toxicity, residue loads and average prevalence in pollen (14–32%) and/or honey (4–47%). Other highly toxic insecticides such as fipronil, and pyrethroids could also have some impact on larvae and nurse bees, but low prevalence of their residues in pollen (usually <5%) and their absence in nectar or honey ensures their risks are low compared to that of neonicotinoids and cholinesterase inhibitors. Despite being designed to stop moulting in insects, average residues of diflubenzuron in pollen (80 ppb) pose little risk to bumble bees under chronic exposure because they are rarely found in that matrix (1% prevalence).

## Conclusions

The large number and frequency of pesticide residues found in pollen and nectar of crop plants pose a clear risk to bee pollinators. Based solely on contact exposure, some 18 compounds (mostly pyrethroids and neonicotinoids) pose a threat to worker bees, but only five insecticides, namely thiamethoxam, phosmet, imidacloprid, chlorpyrifos and clothianidin, and four insecticide-fungicide mixtures pose risks with probabilities above 5%.

Those three neonicotinoids plus the organochlorine lindane pose the highest risk to worker bees and larvae when feeding on contaminated honey or nectar, but only thiamethoxam is of great concern when they feed on contaminated pollen, honey or nectar. In addition, risks of systemic neonicotinoids are probably underestimated because of their time-cumulative toxicity, synergistic effects with ergosterol inhibiting fungicides, and additive effects in combination with pyrethroids. Further research on the combined effects of such mixtures is needed to fully understand the reasons behind the collapse of honey bee and bumble bee colonies.

## Supporting Information

Table S1 **Pesticide residues (μg kg^−1^ or ppb) found in pollen, honey or nectar and wax together with their average prevalence (%) in Europe, the Americas and Asia.**
(DOC)

Table S2 **Acute toxicity (LD50 μg bee^−1^) of pesticides to honey bees and bumble bees.**
(DOC)

Table S3 **Estimated average and maximum daily doses (ng bee^−1^) of pesticide residues ingested by bees – herbicides excluded.**
(DOC)

File SI **Estimation of doses and risks by contact and dietary exposure.**
(DOCX)
