# Supplementary material for: Pesticide Residues and Bees – A Risk Assessment
Source: PLoS One. 2014 Apr 9;9(4):e94482. doi: 10.1371/journal.pone.0094482 (PMC3981812; doi:10.1371/journal.pone.0094482)
Supplement: Table S1 — Pesticide residues (μg kg−1 or ppb) found in pollen, honey or nectar and wax together with their average prevalence (%) in Europe, the Americas and Asia. (DOC) [file pone.0094482.s001.doc]

**Table S1.** Pesticide residues (µg kg^-1^ or ppb) found in pollen, honey or nectar and wax together with their average prevalence (%) in Europe, the Americas and Asia.

|  |  | Pollen |  |  |  | Honey or nectar | |  |  | Wax |  |  |  |
| --- | --- | --- | --- | --- | --- | --- | --- | --- | --- | --- | --- | --- | --- |
| Use^1^ | Chemical^2^ | (%) | Average | Max | Sources^3^ | (%) | Average | Max | Sources^3^ | (%) | Average | Max | Sources^3^ |
| I | acephate | 1.1 | 91.0 | 163.0 | l, s |  |  | 52.0 | i |  |  |  |  |
| I | acetamiprid | 24.1 | 3.0 | 134.0 | l, m | 51.0 | 2.4 | 13.3 | m |  |  |  |  |
| A | acrinathrin (total) | 2.0 | 146.8 | 875.0 | c |  |  | 2400.0 | i | 6.7 | 139.0 | 139.0 | p |
| H | alachlor | 1.0 | 48.1 | 124.0 | s |  |  |  |  |  |  |  |  |
| I-A | aldicarb (total) | 2.5 | 13.0 | 1342.2 | c, l, m |  |  |  |  | 9.1 | 27.4 | 698.6 | l, t |
| I | aldrin |  |  |  |  |  | 10.0 | 150.0 | i, o |  |  |  |  |
| I | allethrin | 1.1 | 0.1 | 11.0 | l |  |  |  |  | 3.8 | 28.0 | 139.0 | l |
| H | amicarbazone | 0.3 | 98.0 | 98.0 | l |  |  |  |  |  |  |  |  |
| A | amitraz (total) | 20.0 | 58.4 | 1117.0 | c, l, n |  |  |  |  | 29.8 | 585.5 | 46820.0 | l, p, t |
| H | atrazine | 12.8 | 20.5 | 104.0 | c, l, n, s |  | 40.0 | 81.0 | o | 13.9 | 8.2 | 31.0 | l |
| I | azinphos methyl | 2.8 | 26.1 | 643.0 | c, l, s | 4.4 | 21.8 | 55.3 | g | 4.0 | 131.7 | 817.0 | g, l |
| F | azoxystrobin | 6.5 | 7.7 | 107.0 | c, l, n, s |  |  | 4.0 | i | 26.8 | 17.2 | 278.0 | l, t |
| I | bendiocarb |  |  |  |  |  |  |  |  | 0.8 | 13.8 | 22.0 | l |
| H | bentazone | 0.6 | 4.9 | 7.2 | s |  |  |  |  |  |  |  |  |
| I | beta-cyfluthrin | 4.0 | 2.2 | 34.0 | l, n |  | 9.0 | 12.8 | o | 10.2 | 47.0 | 158.0 | g, l, t |
| I | bifenthrin | 6.6 | 2.2 | 13.0 | l, n |  |  | 3.0 | j | 12.8 | 9.8 | 56.1 | l |
| F | bitertanol |  |  |  |  |  |  | 0.1 | i |  |  |  |  |
| F | boscalid | 4.3 | 22.5 | 962.0 | l, s |  |  |  |  | 12.6 | 72.4 | 388.0 | l, t |
| H | bromacil | 1.0 | 5.5 | 9.3 | s |  |  |  |  |  |  |  |  |
| I | bromophos ethyl |  |  |  |  |  | 11.5 | 12.7 | d |  |  |  |  |
| A | bromopropylate | 3.9 | 26.8 | 179.0 | c |  | 15.0 | 245.0 | i, o | 87.9 | 16.4 | 194.8 | p |
| F | captan (total) | 7.0 | 820.8 | 10363.0 | c, l, n |  |  | 19.0 | i | 7.9 | 71.6 | 99.0 | l, t |
| I | carbaryl | 16.6 | 58.9 | 1050.0 | c, g, l, s | 1.5 | 23.4 | 42.0 | d, g, i | 0.5 | 4.5 | 4.5 | l |
| F | carbendazim | 13.0 | 52.0 | 1800.0 | l, n, s |  |  | 27.0 | i | 20.6 | 22.1 | 133.0 | l, t |
| I-A | carbofuran (total) | 1.5 | 12.2 | 137.5 | c, g, l, s | 5.7 | 50.6 | 645.0 | d, g | 4.3 | 19.4 | 55.0 | l, t |
| H | carfentrazone ethyl |  |  |  |  |  |  |  |  | 1.0 | 11.0 | 17.0 | l |
| I | chlordane | 0.2 | 17.5 | 23.0 | c |  |  |  |  |  |  |  |  |
| I-A | chlordimeform |  |  |  |  |  |  |  |  | 6.7 | 145.0 | 145.0 | p |
| I | chlorfenapyr | 0.8 | 1.3 | 1.4 | l |  |  |  |  | 1.7 | 5.6 | 11.9 | l |
| I-A | chlorfenvinphos | 12.2 | 36.8 | 420.0 | c, l | 10.0 | 0.2 | 0.2 | b | 95.9 | 1155.8 | 10640.0 | p |
| F | chlorothalonil | 26.7 | 802.3 | 98900.0 | c, l |  | 10.0 | 15.8 | o | 55.6 | 541.8 | 53700.0 | l, t |
| I | chlorpyrifos | 14.3 | 32.6 | 830.0 | c, g, l, n, s | 22.0 | 3.9 | 15.0 | b, o | 33.6 | 54.9 | 890.0 | g, l, t |
| I | clothianidin | 11.0 | 9.4 | 41.2 | h, m, u | 17.0 | 1.9 | 10.1 | h, i, m, u | 7.7 | 35.0 | 35.0 | p, t |
| I-A | coumaphos (total) | 32.6 | 128.3 | 5917.0 | c, g, l, n, s | 47.5 | 105.5 | 2020.0 | b, g, k | 67.7 | 1351.5 | 93200.0 | g, k, l, p, t |
| A | cymiazole |  |  |  |  |  |  | 17.0 | i |  |  |  |  |
| I-A | cypermethrin | 3.0 | 13.9 | 64.0 | c, l, n | 5.9 | 18.1 | 92.0 | f, o | 9.6 | 22.7 | 131.0 | g, l, t |
| F | cyproconazole | 1.7 | 7.5 | 7.5 | g |  |  |  |  |  |  |  |  |
| F | cyprodinil | 3.1 | 13.2 | 344.0 | l, n, s |  |  |  |  | 18.2 | 30.9 | 106.0 | l, t |
| I | DDT (total) | 0.6 | 31.2 | 110.0 | c, l | 13.9 | 44.2 | 658.0 | d, f | 2.4 | 17.2 | 31.0 | l |
| I | deltamethrin | 1.6 | 25.7 | 91.0 | c, g, l | 1.4 | 4.6 | 6.7 | f, g | 2.1 | 112.3 | 613.0 | g, l |
| I | dialifos |  |  |  |  |  |  | 92.0 | i |  |  |  |  |
| I-A | diazinon | 3.1 | 8.5 | 42.0 | c, l, s |  | 17.0 | 35.0 | d, i, o | 8.5 | 1.6 | 4.3 | l, t |
| F | dichlofluanid |  |  |  |  |  | 8.0 | 10.8 | a |  |  |  |  |
| I | dichlorobenzene |  |  |  |  |  |  | 112.0 | i | 3.8 | 228.0 | 1050.0 | l |
| I | dichlorvos | 0.6 | 6.8 | 9.4 | s |  | 8.0 | 13.3 | o |  |  |  |  |
| F | dicloran |  |  |  |  |  |  | 2.0 | i |  |  |  |  |
| A | dicofol | 4.1 | 12.5 | 143.0 | c, l | 2.0 | 2.2 | 90.0 | f, i | 10.1 | 6.8 | 21.0 | l |
| I | dieldrin | 0.6 | 55.5 | 105.0 | c, n |  |  | 13.0 | i | 1.2 | 18.1 | 35.4 | l |
| F | difenoconazole | 1.5 | 69.3 | 214.1 | c, g, l, s |  |  | 0.9 | i |  |  |  |  |
| IGR | diflubenzuron | 1.1 | 79.7 | 128.0 | l, n |  |  |  |  |  |  |  |  |
| I | dimethoate | 1.3 | 2.3 | 4.2 | s | 5.9 | 4.8 | 8.7 | f |  |  |  |  |
| F | dimethomorph | 3.5 | 10.6 | 166.0 | l, s |  |  |  |  | 1.1 | 95.5 | 133.0 | l |
| I | dinotefuran | 1.0 | 45.3 | 168.1 | h, s |  | 13.7 | 21.6 | h | 7.7 | 97.0 | 97.0 | t |
| F | diphenamide | 0.6 | 1.0 | 1.0 | l |  |  |  |  |  |  |  |  |
| F | diphenylamine | 5.8 | 13.2 | 32.0 | l |  |  |  |  | 23.0 | 151.0 | 281.0 | t |
| H | dithiopyr | 18.5 | 8.9 | 199.0 | s |  |  |  |  |  |  |  |  |
| I-A | endosulfan (total) | 10.9 | 77.3 | 3180.0 | c, g, l, n | 15.9 | 20.4 | 27.0 | b, f, o | 28.6 | 61.7 | 255.2 | g, l, p, t |
| I | endrin | 0.8 | 114.0 | 320.0 | c |  |  | 7.0 | i |  |  |  |  |
| I | esfenvalerate | 7.9 | 3.3 | 60.0 | l, n |  |  |  |  | 31.4 | 7.0 | 56.1 | l, t |
| I | ethion |  |  |  |  |  |  |  |  | 1.0 | 107.3 | 131.0 | l |
| H | ethoflumesate |  |  |  |  |  |  |  |  | 1.0 | 392.0 | 560.0 | l |
| A | etoxazole |  |  |  |  |  |  | 1.0 | i |  |  |  |  |
| F | famoxadone | 1.7 | 98.3 | 141.0 | l |  |  |  |  |  |  |  |  |
| F | fenamidone | 0.3 | 73.9 | 73.9 | l |  |  |  |  | 0.5 | 138.0 | 138.0 | l |
| F | fenbuconazole | 3.3 | 100.4 | 396.0 | l, n, s |  |  |  |  | 5.7 | 54.2 | 183.0 | l |
| F | fenhexamid | 2.3 | 51.3 | 182.0 | l, s |  |  |  |  | 4.1 | 27.7 | 46.0 | l, t |
| I | fenitrothion | 0.2 | 5.5 | 7.0 | c |  |  |  |  | 1.1 | 511.0 | 511.0 | g |
| I | fenpropathrin | 8.4 | 35.4 | 170.0 | l, n, s |  |  |  |  | 12.4 | 15.9 | 200.0 | l, t |
| A | fenpyroximate | 11.1 | 28.4 | 114.0 | n |  |  |  |  |  |  |  |  |
| I | fenthion | 5.1 | 41.1 | 197.0 | s |  |  |  |  |  |  |  |  |
| I | fenvalerate |  |  |  |  | 2.0 | 0.7 | 0.7 | f |  |  |  |  |
| I | fipronil (total) | 2.8 | 1.6 | 29.0 | c, g, l, s |  |  |  |  | 1.4 | 12.8 | 35.9 | l |
| I | flumethrin |  |  |  |  |  |  | 1.0 | i | 6.7 | 158.0 | 158.0 | p |
| F | fluoxastrobin |  |  |  |  |  |  |  |  | 1.0 | 33.8 | 44.5 | l |
| H | fluridone |  |  |  |  |  |  |  |  | 1.0 | 6.2 | 6.6 | l |
| F | flusilazole | 1.8 | 14.6 | 71.0 | c, g |  |  | 0.03 | i |  |  |  |  |
| F | flutolanil |  |  |  |  |  |  |  |  | 1.9 | 55.2 | 105.0 | l |
| I | fonofos |  |  |  |  |  | 15.0 | 17.2 | d |  |  |  |  |
| I-A | gamma-HCH (lindane) | 1.2 | 7.6 | 23.0 | c, g | 22.8 | 176.5 | 4310.0 | d, f, s | 2.3 | 18.8 | 32.2 | g |
| I | HCH (a and b) |  |  |  |  | 26.9 | 111.1 | 3530.0 | d, f |  |  |  |  |
| I | heptachlor | 0.5 | 74.5 | 120.0 | c |  |  | 57.0 | i | 2.4 | 18.6 | 44.3 | g, l |
| I | heptenophos |  |  |  |  | 16.0 | 80.0 | 230.0 | d |  |  |  |  |
| F | hexachlorobenzene | 1.6 | 0.9 | 4.0 | c, l | 32.0 | 25.0 | 270.0 | d, o | 0.4 | 1.0 | 1.0 | l |
| F | hexaconazole | 3.3 | 54.7 | 106.0 | g |  |  |  |  |  |  |  |  |
| F | imazalil | 0.3 | 1.0 | 1.0 | s |  |  |  |  |  |  |  |  |
| H | imazamethazir | 0.4 | 9.5 | 13.0 | c |  |  |  |  |  |  |  |  |
| I | imidacloprid (total) | 16.2 | 19.7 | 912.0 | g, h, l, n, s | 21.4 | 6.0 | 72.8 | e, g, h, m, o, q, r, s | 4.3 | 26.5 | 45.0 | l, t |
| I | indoxacarb | 2.2 | 108.4 | 417.0 | l, s |  |  |  |  |  |  |  |  |
| F | iprodione | 0.2 | 3.5 | 10.0 | c, l |  |  |  |  | 7.2 | 276.4 | 636.0 | l, t |
| I | lambda-cyhalothrin | 6.2 | 7.1 | 36.2 | c, l, n | 3.9 | 0.7 | 0.8 | f, o | 5.0 | 6.5 | 16.9 | l, t |
| I-A | malathion | 1.6 | 17.1 | 70.0 | c, l, s | 5.9 | 98.0 | 243.0 | f | 3.9 | 68.8 | 184.0 | l, p |
| F | metalaxyl | 1.7 | 14.7 | 37.9 | c, n, s |  |  |  |  | 0.5 | 1.4 | 1.4 | l |
| I | methamidophos | 0.3 | 22.0 | 22.0 | s |  |  |  |  |  |  |  |  |
| I | methidathion | 4.0 | 0.9 | 33.0 | l | 8.0 | 40.0 | 68.0 | d | 5.8 | 15.3 | 78.7 | l |
| I | methiocarb | 0.3 | 1.4 | 1.4 | s | 14.0 | 15.0 | 27.0 | d |  |  |  |  |
| I-A | methomyl | 3.8 | 10.3 | 24.0 | s |  |  |  |  |  |  |  |  |
| I | methoxychlor |  |  |  |  |  |  | 593.0 | i |  |  |  |  |
| I | methoxyfenozide | 8.3 | 2.9 | 128.0 | l |  |  | 3.0 | i | 18.8 | 81.5 | 495.0 | l |
| H | metolachlor | 8.4 | 2.1 | 103.0 | l, s |  |  |  |  |  |  |  |  |
| H | metribuzin | 5.1 | 0.3 | 44.0 | l |  |  |  |  | 1.0 | 4.5 | 8.0 | l |
| I | mevinphos |  |  |  |  |  |  |  |  | 3.9 | 138.0 | 204.0 | g |
| F | myclobutanil | 2.4 | 159.6 | 4190.0 | c, g, l, s |  |  |  |  |  |  |  |  |
| H | napropamide | 3.2 | 6.3 | 29.7 | s |  |  |  |  |  |  |  |  |
| H | norflurazon | 5.1 | 1.5 | 108.0 | l |  |  |  |  | 7.0 | 5.4 | 38.1 | l, t |
| H | oxadiazon | 0.3 | 6.2 | 6.2 | s |  |  |  |  |  |  |  |  |
| I-A | oxamyl | 0.8 | 30.9 | 49.0 | c, g, l |  |  |  |  | 7.7 | 22.0 | 22.0 | t |
| H | oxyfluorfen | 1.5 | 4.7 | 18.0 | l, n, s |  |  |  |  | 14.6 | 6.6 | 34.0 | l, t |
| I-A | parathion | 0.4 | 14.1 | 19.2 | c, g |  |  |  |  | 1.1 | 99.0 | 99.0 | g |
| I | parathion methyl | 2.0 | 24.8 | 24.8 | g | 2.0 | 10.0 | 50.0 | d, i | 1.4 | 4.6 | 6.1 | l |
| F | penconazole | 5.5 | 17.6 | 126.0 | g |  |  |  |  |  |  |  |  |
| H | pendimethalin | 14.9 | 31.6 | 1730.0 | c, l, n, s |  |  |  |  | 27.8 | 10.9 | 84.0 | l |
| I | permethrin | 1.8 | 10.5 | 92.0 | l, n |  |  | 27.0 | i | 4.8 | 156.3 | 372.0 | l, t |
| I | phenothrin | 0.3 | 83.9 | 83.9 | l |  |  |  |  |  |  |  |  |
| I | phorate |  |  |  |  | 24.0 | 0.2 | 0.9 | b |  |  |  |  |
| I | phosalone | 0.4 | 31.3 | 31.3 | l |  |  |  |  | 7.7 | 32.0 | 32.0 | t |
| I | phosmet | 13.2 | 339.3 | 16556.0 | l, n, s |  |  |  |  | 1.9 | 69.0 | 209.0 | l |
| IS | piperonyl butoxide |  |  |  |  |  |  | 10.0 | i | 1.0 | 119.6 | 208.0 | l |
| I | pirimicarb |  |  |  |  | 4.0 | 38.0 | 71.0 | d |  |  |  |  |
| I | pirimiphos ethyl |  |  |  |  |  | 19.0 | 22.0 | d |  |  |  |  |
| I | prallethrin | 0.9 | 6.5 | 7.6 | l |  |  |  |  | 1.0 | 5.6 | 6.8 | l |
| F | procymidone | 1.8 | 30.6 | 87.0 | c |  |  |  |  | 1.3 | 27.7 | 27.7 | g |
| H | prodiamine | 0.3 | 9.5 | 9.5 | s |  |  |  |  |  |  |  |  |
| H | pronamide | 3.1 | 3.9 | 378.0 | l |  |  |  |  | 4.8 | 6.1 | 22.8 | l |
| H | propanyl | 0.6 | 311.5 | 358.0 | l |  |  |  |  |  |  |  |  |
| F | propiconazole | 1.8 | 5.5 | 361.0 | c, l, s |  |  |  |  | 1.0 | 196.5 | 227.0 | l |
| I | propoxur | 0.3 | 7.5 | 7.5 | s |  |  |  |  |  |  |  |  |
| H | propyzamide | 0.6 | 83.0 | 94.0 | s |  |  |  |  |  |  |  |  |
| F | pyraclostrobin | 1.1 | 25.5 | 265.0 | l, n, s |  |  | 17.0 | i | 4.8 | 84.2 | 438.0 | l |
| I | pyrazophos |  |  |  |  |  | 6.2 | 7.4 | d |  |  |  |  |
| I | pyrethrins | 0.9 | 0.4 | 62.0 | l |  |  |  |  | 7.0 | 156.8 | 229.0 | l, t |
| A | pyridaben | 1.4 | 18.8 | 26.6 | l |  |  |  |  | 0.5 | 5.4 | 5.4 | l |
| F | pyrimethanil | 3.5 | 14.2 | 83.0 | l, s |  |  | 4.0 | i | 1.4 | 14.3 | 27.8 | l |
| I | pyriproxyfen |  |  |  |  |  |  |  |  | 1.0 | 4.9 | 7.6 | l |
| I | quinalphos |  |  |  |  | 3.9 | 9.6 | 10.3 | f |  |  |  |  |
| F | quintozene |  |  |  |  |  |  |  |  | 1.4 | 1.6 | 2.5 | l |
| I-A | rotenone | 0.1 | 23.0 | 23.0 | c |  |  |  |  |  |  |  |  |
| H | sethoxydim | 0.3 | 173.0 | 173.0 | l |  |  | 8.0 | i |  |  |  |  |
| H | simazine | 3.3 | 18.3 | 101.0 | c, l, s |  | 16.0 | 17.0 | o |  |  |  |  |
| A | spirodiclofen |  |  |  |  |  |  |  |  | 0.5 | 28.5 | 28.5 | l |
| I | spiromesifen | 0.3 | 34.3 | 63.0 | c, l |  |  |  |  |  |  |  |  |
| H | sulfometuron-methyl | 0.3 | 37.0 | 37.0 | s |  |  |  |  |  |  |  |  |
| I-A | tau-fluvalinate | 32.2 | 123.4 | 2670.0 | c, g, l, n, s | 0.9 | 15.9 | 750.0 | g, i | 82.8 | 3143.6 | 204000.0 | g, k, l, p, t |
| F | tebuconazole | 2.8 | 16.5 | 33.2 | g |  | 4.0 | 5.0 | o |  |  |  |  |
| I | tebufenozide | 7.7 | 1.1 | 58.0 | l |  |  |  |  | 4.8 | 8.0 | 27.7 | l |
| H | tebuthiuron | 2.3 | 0.4 | 48.0 | l |  |  |  |  | 1.4 | 11.0 | 22.4 | l |
| I | tefluthrin |  |  |  |  |  |  |  |  | 0.5 | 3.3 | 3.3 | l |
| I | temephos |  |  |  |  |  | 7.2 | 8.1 | d |  |  |  |  |
| A | tetradifon | 4.3 | 14.7 | 243.0 | c |  | 14.0 | 23.4 | o | 1.0 | 7.9 | 11.1 | l |
| I | tetramethrin | 0.3 | 6.1 | 6.1 | l |  |  |  |  |  |  |  |  |
| F | thiabendazole | 1.7 | 1.2 | 6.0 | l, s |  |  |  |  | 1.4 | 34.1 | 76.0 | l |
| I | thiacloprid | 17.7 | 75.1 | 1002.2 | l, m, n, s | 64.0 | 6.5 | 208.8 | i, m | 4.8 | 59.2 | 113.0 | l, t |
| I | thiamethoxam | 12.8 | 28.9 | 127.0 | h, l, m, s, u | 65.0 | 6.4 | 17.0 | h, m, s, u | 7.7 | 38.0 | 38.0 | t |
| F | thiophanate-methyl | 8.9 | 110.9 | 1413.0 | s |  |  |  |  |  |  |  |  |
| F | thymol | 27.3 | 2271.8 | 39700.0 | n |  |  |  |  |  |  |  |  |
| F | triadimefon |  |  |  |  |  |  |  |  | 0.5 | 2.4 | 2.4 | l |
| H | triallate |  |  |  |  |  | 4.4 | 4.4 | a |  |  |  |  |
| I | tribufos | 0.3 | 3.5 | 3.5 | l |  |  |  |  | 2.9 | 25.1 | 59.0 | l |
| I | trichlorfon | 0.3 | 14.0 | 14.0 | s |  |  |  |  |  |  |  |  |
| F | trifloxystrobin | 5.3 | 14.0 | 264.0 | l, s |  |  | 0.3 | i | 3.1 | 6.7 | 22.4 | l |
| H | trifluralin | 4.7 | 1.5 | 23.0 | c, l, n |  | 9.0 | 13.8 | o | 12.5 | 3.9 | 36.0 | l |
| F | vinclozolin | 0.8 | 2.0 | 7.0 | c, l | 11.7 | 74.8 | 132.0 | g, j, o | 4.6 | 10.4 | 27.0 | g, l, t |
|  |  |  |  |  |  |  |  |  |  |  |  |  |  |
|  | Highest | 32.6 | 2271.8 | 98900.0 |  | 65.0 | 176.5 | 4310.0 |  | 95.9 | 3143.6 | 204000.0 |  |
|  | Average | 4.9 | 66.3 | 1654.0 |  | 16.6 | 24.7 | 232.0 |  | 10.4 | 125.7 | 4441.2 |  |
|  | Median | 2.1 | 15.6 | 93.0 |  | 10.9 | 11.5 | 17.0 |  | 4.3 | 27.4 | 76.0 |  |
|  | Lowest | 0.1 | 0.1 | 1.0 |  | 0.9 | 0.2 | 0.03 |  | 0.4 | 1.0 | 1.0 |  |

^1^ A = acaricide ; F = fungicide ; H = herbicide ; I = insecticide ; IGR = insect growth regulator ; IS = insecticide synergist

^2^ Total residues = parent compound + toxic metabolites

^3^ Sources: a = Albero et al. 2004 ; b = Balayiannis et al. 2008 ; c = Bernal et al. 2010 ; d = Blasco et al. 2003 & 2008 ; e = Byrne et al. 2013 ; f = Choudhary & Sharma 2008 ; g = Chauzat et al. 2011 ; h = Dively et al. 2010 ; i = Johnson et al. 2010 ; j = Kubik et al. 1999 ; k = Martel et al. 2007 ; l = Mullin et al. 2010 ; m = Pohorecka et al. 2012 ; n = Rennich et al. 2012 ; o = Rissato et al. 2004 & 2007 ; p = Serra-Bonhevi & Orantes-Bermejo 2010 ; q = Schmuck et al. 2001 ; r = Scott-Dupree & Spivak 2001 ; s = Stoner & Eitzer 2012 & 2013 ; t = Wu et al. 2011 ; u = Pilling et al. 2014.
