# Supplementary material for: Pesticide Residues and Bees – A Risk Assessment
Source: PLoS One. 2014 Apr 9;9(4):e94482. doi: 10.1371/journal.pone.0094482 (PMC3981812; doi:10.1371/journal.pone.0094482)
Supplement: Table S2 — Acute toxicity (LD50 μg bee−1) of pesticides to honey bees and bumble bees. (DOC) [file pone.0094482.s002.doc]

**Table S2.** Acute toxicity (LD50 µg bee^-1^) of pesticides to honey bees and bumble bees^1^

| CAS-RN | Chemical | Use^2^ | *Apis mellifera* | | *Bombus* spp. | |
| --- | --- | --- | --- | --- | --- | --- |
|  |  |  | Contact | Oral | Contact | Oral |
| 3547-33-9 | 2-(octylthio)ethanol | IR | 57 |  |  |  |
| 30560-19-1 | acephate | I | 1.8 | 0.23 | 4.0 | 7.9 |
| 57960-19-7 | acequinocyl | A | 280 | 315 |  |  |
| 135410-20-7 | acetamiprid | I | 7.9 | 14 | 100 | 22 |
| 101007-06-1 | acrinathrin | A | 0.17 | 0.12 |  |  |
| 83130-01-2 | alanycarb | I | 0.67 |  |  |  |
| 116-06-3 | aldicarb | I-A | 0.38 |  |  |  |
| 67375-30-8 | alpha-cypermethrin | I | 0.044 | 0.059 |  |  |
| 348635-87-0 | amisulbrom | F | >100 | >100 |  |  |
| 33089-61-1 | amitraz | A | 50 |  |  |  |
| 65195-55-3 | abamectin | I | 0.030 |  | 0.14 | 0.07 |
| 11141-17-6 | azadirachtin | I | 6.1 | 3.7 |  |  |
| 35575-96-3 | azamethiphos | I | 10 | 0.1 |  |  |
| 41083-11-8 | azocyclotin | A | >100 |  |  |  |
| 131860-33-8 | azoxystrobin | F | >200 | >25 |  |  |
| 71626-11-4 | benalaxyl | F | >100 |  |  |  |
| 98243-83-5 | benalaxyl-M | F | >104 | >104 |  |  |
| 22781-23-3 | bendiocarb | I | 0.43 | 0.52 |  |  |
| 82560-54-1 | benfuracarb | I | 0.16 |  |  |  |
| 17804-35-2 | benomyl | F | >50 |  |  |  |
| 17606-31-4 | bensultap | I | 26 |  |  |  |
| 177406-68-7 | benthiavalicarb-isopropyl | F | >100 | >100 |  |  |
| 68359-37-5 | beta-cyfluthrin | I | 0.031 | 0.05 | 0.46 | 0.12 |
| 52315-07-8 | beta-cypermethrin | I |  | 1.8 |  |  |
| 149877-41-8 | bifenazate | A | 8.3 | 141 |  |  |
| 82657-04-3 | bifenthrin | I | 0.015 | 0.20 |  | 0.34 |
| 584-79-2 | bioallethrin | I | 5.35 | 6.85 |  |  |
| 28434-01-7 | bioresmethrin | I | 0.019 | 0.002 |  |  |
| 201593-84-2 | bistrifluron | I | >100 |  |  |  |
| 55179-31-2 | bitertanol | F | >200 | 104 |  |  |
| 8011-63-0 | Bordeaux mixture (Cu) | F | 25 | 23 |  |  |
| 188425-85-6 | boscalid | F | >200 | 166 |  |  |
| 18181-80-1 | bromopropylate | A | 183 |  |  |  |
| 116255-48-2 | bromuconazole | F | >500 | >100 |  |  |
| 41483-43-6 | bupirimate | F | >500 | 632 |  |  |
| 69327-76-0 | buprofezin | IGR | 20 |  |  | 23* |
| 34681-10-2 | butocarboxim | I | 1 |  |  |  |
| 2425-06-1 | captafol | F | 97 |  |  |  |
| 133-06-2 | captan | F | 215 | 91 |  |  |
| 63-25-2 | carbaryl | I | 0.84 | 0.15 | 41 | 3.9 |
| 10605-21-7 | carbendazim | F | >50 |  |  |  |
| 1563-66-2 | carbofuran | I-A | 0.16 |  |  |  |
| 55285-14-8 | carbosulfan | I | 0.28 | 1.1 |  |  |
| 5234-68-4 | carboxin | F | 181 |  |  |  |
| 22042-59-7 | cartap hydrochloride | I |  |  |  | 2.2 |
| 2439-01-2 | chinomethionat | F | 81 |  |  |  |
| 500008-45-7 | chlorantraniliprole | I | 4 | 104 |  |  |
| 57-74-9 | chlordane | I | 0.6 |  |  |  |
| 54593-83-8 | chlorethoxyfos | I | 0.09 |  |  |  |
| 122453-73-0 | chlorfenapyr | I | 0.15 |  |  |  |
| 470-90-6 | chlorfenvinphos | I-A | 4.1 | 0.55 |  |  |
| 71422-67-8 | chlorfluazuron | IGR |  | >100 |  |  |
| 1897-45-6 | chlorothalonil | F | 135 | 63 |  |  |
| 2921-88-2 | chlorpyrifos | I | 0.072 | 0.24 |  |  |
| 5598-13-0 | chlorpyrifos-methyl | I | 0.28 | 0.11 | 0.09 | 0.23 |
| 143807-66-3 | chromafenozide | I | >100 | 133 |  |  |
| 67564-91-4 | cis-fenpropimorph | F | >100 | 95 |  |  |
| 74115-24-5 | clofentezine | A | 48 | 71 |  |  |
| 210880-92-5 | clothianidin | I | 0.039 | 0.0035 | 0.016 |  |
| 56-72-4 | coumaphos | I-A | 20 | 4.6 |  |  |
| 120116-88-3 | cyazofamid | F | >100 | 152 |  |  |
| 560121-52-0 | cyenopyrafen | A | >100 | >100 |  |  |
| 180409-60-3 | cyflufenamid | F | >100 | >100 |  |  |
| 400882-07-7 | cyflumetofen | A | 102 | 590 |  |  |
| 68359-37-5 | cyfluthrin | I | 0.019 | 0.051 |  |  |
| 68085-85-8 | cyhalothrin | I | 0.022 |  |  |  |
| 13121-70-5 | cyhexatin | A | 34 |  |  |  |
| 57966-95-7 | cymoxanil | F | >25 | 51 |  |  |
| 52315-07-8 | cypermethrin | I-A | 0.034 | 0.064 |  |  |
| 94361-06-5 | cyproconazole | F | >100 | >1000 |  |  |
| 121552-61-2 | cyprodinil | F | 280 | >100 |  |  |
| 66215-27-8 | cyromazine | IGR | 35 | 20 | 2.5* | 2.9* |
| 533-74-4 | dazomet | B | 34 | >10 |  |  |
| 52918-63-5 | deltamethrin | I | 0.024 | 0.27 | 0.28 |  |
| 919-86-8 | demeton-S-methyl | I | 0.56 | 0.67 |  |  |
| 80060-09-9 | diafenthiuron | I | 1.5 | 2.1 |  |  |
| 333-41-5 | diazinon | I-A | 0.38 | 0.21 |  |  |
| 62-73-7 | dichlorvos | I | 0.46 | 0.29 |  |  |
| 62865-36-5 | diclomezine | F | >100 | >100 |  |  |
| 99-30-9 | dicloran | F | 180 |  |  |  |
| 115-32-2 | dicofol | A | 19 | 10 |  |  |
| 141-66-2 | dicrotophos | I | 0.036 | 0.13 |  |  |
| 87130-20-9 | diethofencarb | F | 20 |  |  |  |
| 119446-68-3 | difenoconazole | F | 100 | 182 |  |  |
| 43222-48-6 | difenzoquat metilsulfate | F | 36 |  |  |  |
| 162320-67-4 | diflovidazin | A | 25 | 25 |  |  |
| 35367-38-5 | diflubenzuron | IGR | 114 | >100 | 0.1* | 1.5* |
| 130339-07-0 | diflumetorim | F | 29 | >10 |  |  |
| 60-51-5 | dimethoate | I | 0.12 | 0.17 | 1.2 | 0.82 |
| 110488-70-5 | dimethomorph | F | 55 | 32 |  |  |
| 149961-52-4 | dimoxystrobin | F | >100 | 79 |  |  |
| 83657-24-3 | diniconazole | F | >20 |  |  |  |
| 83657-18-5 | diniconazole-M | F | >20 |  |  |  |
| 131-72-6 | dinocap | F | 85 | 90 |  |  |
| 165252-70-0 | dinotefuran | I | 0.049 | 0.022 |  |  |
| 298-04-4 | disulfoton | I | 3.7 | 48 |  |  |
| 3347-22-6 | dithianon | F | >100 |  |  |  |
| 534-52-1 | DNOC | B | 2040 |  |  |  |
| 1593-77-7 | dodemorph | F | >100 | 139 |  |  |
| 31717-87-0 | dodemorph acetate | F | >100 | 139 |  |  |
| 2439-10-3 | dodine | F | 145 |  |  |  |
| 155569-91-8 | emamectin benzoate | I | 0.004 |  |  |  |
| 115-29-7 | endosulfan | I-A | 6.3 | 20 |  |  |
| 2104-64-5 | EPN | I | 0.25 |  |  |  |
| 106325-08-0 | epoxiconazole | F | >100 |  |  |  |
| 66230-04-4 | esfenvalerate | I | 0.026 |  |  |  |
| 162650-77-3 | ethaboxam | F | >100 |  |  |  |
| 563-12-2 | ethion | A | 11 |  |  |  |
| 13194-48-4 | ethoprophos | I | 4.8 |  |  |  |
| 80844-07-1 | etofenprox | I | 0.015 | 0.024 |  |  |
| 153233-91-1 | etoxazole | A | >200 | >200 |  |  |
| 131807-57-3 | famoxadone | F | >25 | 51 |  |  |
| 161326-34-7 | fenamidone | F | 64 | 159 |  |  |
| 22224-92-6 | fenamiphos | B | 0.72 | 0.45 |  |  |
| 60168-88-9 | fenarimol | F | >100 | 23 |  |  |
| 120928-09-8 | fenazaquin | A | 7.4 | 8.9 |  |  |
| 114369-43-6 | fenbuconazole | F | 290 |  |  |  |
| 13356-08-6 | fenbutatin oxide | A | >200 | >200 |  |  |
| 126833-17-8 | fenhexamid | F | 207 | 1.7 |  |  |
| 122-14-5 | fenitrothion | I | 0.52 |  | 0.76 |  |
| 62850-32-2 | fenothiocarb | A | 300 |  |  |  |
| 72490-01-8 | fenoxycarb | I | >100 | 36 |  |  |
| 67306-00-7 | fenpropidin | F | 46 | >10 |  |  |
| 67306-03-0 | fenpropimorph | F | >100 | >100 |  |  |
| 134098-61-6 | fenpyroximate | A | 11 |  |  |  |
| 55-38-9 | fenthion | I | 0.22 |  |  |  |
| 76-87-9 | fentin hydroxide | F | 115 |  |  |  |
| 51630-58-1 | fenvalerate | I | 1.0 |  |  |  |
| 14484-64-1 | ferbam | F | 12 |  |  |  |
| 120068-37-3 | fipronil | I | 0.007 | 0.001 |  |  |
| 158062-67-0 | flonicamid | I | >100 | 44 |  |  |
| 229977-93-9 | fluacrypyrim | A | >10 | 15 |  |  |
| 79622-59-6 | fluazinam | F | 143 | >100 |  |  |
| 272451-65-7 | flubendiamide | I | >200 | >200 |  |  |
| 70124-77-5 | flucythrinate | I | 0.3 |  |  |  |
| 131341-86-1 | fludioxonil | F | 50 | 329 |  |  |
| 101463-69-8 | flufenoxuron | IGR | >100 | 109 | 0.67* | 2.8* |
| 69770-45-2 | flumethrin | I | 0.05 |  |  |  |
| 211867-47-9 | flumorph | F | 170 |  |  |  |
| 239110-15-7 | fluopicolide | F | >100 | 241 |  |  |
| 41205-21-4 | fluoroimide | F | 67 | 36 |  |  |
| 361377-29-9 | fluoxastrobin | F | >200 | 843 |  |  |
| 85509-19-9 | flusilazole | F | 150 |  |  |  |
| 106917-52-6 | flusulfamide | F | 182 | 34 |  |  |
| 66332-96-5 | flutolanil | F | 11 | 209 |  |  |
| 76674-21-0 | flutriafol | F | 72 | 31 |  |  |
| 133-07-3 | folpet | F | 49 | 236 |  |  |
| 22259-30-9 | formetanate hydrochloride | A | 3.8 | 1.2 |  |  |
| 15845-66-6 | fosetyl | F | >1000 | 462 |  |  |
| 39148-24-8 | fosetyl aluminium | F | 316 | 462 |  |  |
| 98886-44-3 | fosthiazate | B | 0.26 | 0.61 |  |  |
| 3878-19-1 | fuberidazole | F | >200 | 187 |  |  |
| 57764-08-6 | furalaxyl-M | F |  | 5 |  |  |
| 98-01-1 | furfural | B | 90 | >100 |  |  |
| 76703-62-3 | gamma-cyhalothrin | I | 0.008 | 2.3 |  |  |
| 58-89-9 | gamma-HCH (lindane) | I-A | 0.66 | 0.048 |  |  |
| 108173-90-6 | guazatine | F | >200 |  |  |  |
| 7345-69-9 | GY-81 | F | >25 |  |  |  |
| 111872-58-3 | halfenprox | A | 0.027 |  |  |  |
| 112226-61-6 | halofenozide | I | >100 |  |  |  |
| 23560-59-0 | heptenophos | I |  |  | 2.7 | 0.53 |
| 79983-71-4 | hexaconazole | F | >100 | >100 |  |  |
| 86479-06-3 | hexaflumuron | IGR | 89 | 89 |  |  |
| 78587-05-0 | hexythiazox | A | >200 |  |  |  |
| 67485-29-4 | hydramethylnon | I | 45 |  |  |  |
| 123-33-1 | hydrazide | IGR | >100 | >100 |  |  |
| 41205-09-8 | hydroprene | I | >1000 | 0.1 |  |  |
| 10004-44-1 | hymexazol | F | >100 | >100 |  |  |
| 73790-28-0 | imazalil | F | 39 | 37 |  |  |
| 86598-92-7 | imibenconazole | F | >200 | 125 |  |  |
| 138261-41-3 | imidacloprid | I | 0.061 | 0.013 | 0.02 | 0.027 |
| 57520-17-9 | iminoctadine triacetate | F | >100 | >100 |  |  |
| 72963-72-5 | imiprothrin | I | 0.52 |  |  |  |
| 144171-61-9 | indoxacarb | I | 0.59 | 16 |  |  |
| 26087-47-8 | iprobenfos | F | 37 |  |  |  |
| 36734-19-7 | iprodione | F | 400 | 25 |  |  |
| 140923-17-7 | iprovalicarb | F | >200 | 199 |  |  |
| 283159-90-0 | IR-5885 | F | >100 | 107 |  |  |
| 18854-01-8 | isoxathion | I | 0.082 |  |  |  |
| 19408-46-9 | kasugamycin | F | >40 |  |  |  |
| 42588-37-4 | kinoprene | I | 35 |  |  |  |
| 143390-89-0 | kresoxim-methyl | F | 22 | 14 |  |  |
| 91465-08-6 | lambda-cyhalothrin | I | 0.048 | 0.84 | 0.16 | 0.17 |
| 103055-07-8 | lufenuron | IGR | >200 | 197 |  | 69* |
| 121-75-5 | malathion | I-A | 0.47 | 9.2 |  |  |
| 8018-01-7 | mancozeb | F | 226 | 171 |  |  |
| 374726-62-2 | mandipropamid | F | >200 | >200 |  |  |
| 12427-38-2 | maneb | F | 12 | 173 |  |  |
| 185676-84-0 | MB-599 | IS | 80 | >100 |  |  |
| 110235-47-7 | mepanipyrim | F | >100 | 51 |  |  |
| 55814-41-0 | mepronil | F | >1000 | >100 |  |  |
| 139968-49-3 | metaflumizone | I | 9.4 | 2.4 |  |  |
| 57837-19-1 | metalaxyl | F | 141 | 269 |  |  |
| 70630-17-0 | metalaxyl-M | F | 25 | >25 |  |  |
| 125116-23-6 | metconazole | F | >100 | 87 |  |  |
| 10265-92-6 | methamidophos | I | 0.97 | 0.2 |  |  |
| 950-37-8 | methidathion | I | 0.27 |  |  |  |
| 2032-65-7 | methiocarb | B | 0.29 | 0.47 |  |  |
| 16752-77-5 | methomyl | I | 0.49 | 0.24 |  | 3.4 |
| 40596-69-8 | methoprene | I | >1000 | 0.2 |  |  |
| 72-43-5 | methoxychlor | I | 20 | 5.0 |  |  |
| 161050-58-4 | methoxyfenozide | I | >100 | >100 |  |  |
| 9006-42-2 | metiram | F | 187 | 80 |  |  |
| 133408-50-1 | metominostrobin | F | >100 |  |  |  |
| 220899-03-6 | metrafenone | F | >100 | 114 |  |  |
| 7786-34-7 | mevinphos | I | 0.094 |  |  |  |
| 51596-10-2 & 11-3 | milbemectin | A | 0.025 | 0.43 |  |  |
| 67527-71-3 | mildiomycin | F | >100 | >100 |  |  |
| 6923-22-4 | monocrotophos | I | 0.13 | 0.03 |  |  |
| 88671-89-0 | myclobutanil | F | >40 | 34 |  |  |
| 142-59-6 | nabam | F | 12 |  |  |  |
| 300-76-5 | naled | I | 0.25 |  |  |  |
| 10552-74-6 | nitrothal-isopropyl | F |  | >100 |  |  |
| 116714-46-6 | novaluron | IGR | 122 | >100 | 0.044* | 1.1* |
| 121451-02-3 | noviflumuron | IGR | >100 | >100 |  |  |
| 63284-71-9 | nuarimol | F | >11 |  |  |  |
| 58810-48-3 | ofurace | F |  | >58 |  |  |
| 1113-02-6 | omethoate | I |  | 0.05 |  |  |
| 248593-16-0 | orysastrobin | F | >1420 |  |  |  |
| 77732-09-3 | oxadixyl | F | >100 | >200 |  |  |
| 23135-22-0 | oxamyl | I-A | 0.88 | 0.38 |  |  |
| 5259-88-1 | oxycarboxin | F | 181 | 181 |  |  |
| 301-12-2 | oxydemeton-methyl | I | 7.4 |  |  |  |
| 50-29-3 | p'-p' DDT | I | 8.8 | 5.1 |  |  |
| 56-38-2 | parathion | I-A | 0.60 | 0.04 |  |  |
| 298-00-0 | parathion-methyl | I | 2.7 | 750 |  |  |
| 101903-30-4 | pefurazoate | F | >100 |  |  |  |
| 66246-88-6 | penconazole | F | 12 | 24 |  |  |
| 66063-05-6 | pencycuron | F | >100 | 99 |  |  |
| 87-86-5 | pentachlorophenol | B | 48 |  |  |  |
| 183675-82-3 | penthiopyrad | F | 312 | 385 |  |  |
| 52645-53-1 | permethrin | I | 0.063 | 0.13 | 0.22 |  |
| 26002-80-2 | phenothrin | I | 0.13 | 0.16 |  |  |
| 2597-03-7 | phenthoate | I | 0.31 |  |  |  |
| 62-38-4 | phenylmercury acetate | F |  | 21 |  |  |
| 298-02-2 | phorate | I | 6.0 | 0.44 |  |  |
| 2310-17-0 | phosalone | I |  |  | 5.2 | 4.0 |
| 732-11-6 | phosmet | I | 0.62 | 0.37 |  |  |
| 13171-21-6 | phosphamidon | I | 1.5 |  |  |  |
| 27355-22-2 | phthalide | F | >400 |  |  |  |
| 117428-22-5 | picoxystrobin | F | >200 | >200 |  |  |
| 3478-94-2 | piperalin | F | 36 |  |  |  |
| 51-03-6 | piperonyl butoxide | IS | 17 |  |  |  |
| 23103-98-2 | pirimicarb | I | 36 | 3.8 |  |  |
| 29232-93-7 | pirimiphos-methyl | I | 0.27 | 0.22 |  |  |
| 22976-86-9 | polyoxorim | F |  | >29 |  |  |
| 23031-36-9 | prallethrin | I | 0.028 |  |  |  |
| 67747-09-5 | prochloraz | F | 50 | 60 |  |  |
| 41198-08-7 | profenofos | I | 0.32 |  |  |  |
| 25606-41-1 | propamocarb | F | >100 | 99 |  |  |
| 2312-35-8 | propargite | A | 62 | >100 |  |  |
| 60207-90-1 | propiconazole | F | 50 | 77 |  |  |
| 12071-83-9 | propineb | F | 164 | >70 |  |  |
| 114-26-1 | propoxur | I | 0.51 |  |  |  |
| 189278-12-4 | proquinazid | F | 197 | 125 |  |  |
| 178928-70-6 | prothioconazole | F | 165 | 104 |  |  |
| 123312-89-0 | pymetrozine | I | >200 | 117 |  |  |
| 89784-60-1 | pyraclofos | I | 0.95 |  |  |  |
| 175013-18-0 | pyraclostrobin | F | >100 | 73 |  |  |
| 13457-18-6 | pyrazophos | F | 0.25 |  |  |  |
| 121-21-1 | pyrethrin I | I | 0.14 | 0.10 |  |  |
| 121-29-9 | pyrethrin II | I | 0.33 | 0.022 |  |  |
| 8003-34-7 | pyrethrum | I | 0.18 | 0.057 |  |  |
| 96489-71-3 | pyridaben | I | 0.053 | 0.55 |  |  |
| 179101-81-6 | pyridalyl | I | 50 | >100 |  |  |
| 119-12-0 | pyridaphenthion | I | 0.08 |  |  |  |
| 88283-41-4 | pyrifenox | F | 70 | 59 |  |  |
| 53112-28-0 | pyrimethanil | F | >100 | >100 |  |  |
| 105779-78-0 | pyrimidifen | A | 0.66 | 0.64 |  |  |
| 95737-68-1 | pyriproxyfen | I | >100 |  |  |  |
| 57369-32-1 | pyroquilon | F | >1000 | >20 |  |  |
| 13593-03-8 | quinalphos | I | 0.44 | 0.07 | 0.04 | 0.18 |
| 124495-18-7 | quinoxyfen | F | 79 | 316 |  |  |
| 82-68-8 | quintozene | F | 70 |  |  |  |
| 10453-86-8 | resmethrin | I | 0.030 | 0.069 |  |  |
| 83-79-4 | rotenone | I-A | 0.24 |  |  | 0.83 |
| 8051-02-3 | sabadilla (veratrine) | I | 12 |  |  |  |
| 105024-66-6 | silafluofen | I |  | 0.5 |  |  |
| 175217-20-6 | silthiofam | F | >100 | >104 |  |  |
| 168316-95-8 | spinosad | I | 0.003 | 0.057 | 0.36 |  |
| 148477-71-8 | spirodiclofen | A | 256 | 252 |  |  |
| 283594-90-1 | spiromesifen | I | >200 | 790 |  |  |
| 203313-25-1 | spirotetramat | I | 242 | 195 |  |  |
| 118134-30-8 | spiroxamine | F | 4.2 | 92 |  |  |
| 162320-67-4 | SZI-121 | A | 25 |  |  |  |
| 102851-06-9 | tau-fluvalinate | I-A | 8.7 | 45 |  |  |
| 107534-96-3 | tebuconazole | F | >200 | 83 |  |  |
| 112410-23-8 | tebufenozide | I | 234 |  |  |  |
| 119168-77-3 | tebufenpyrad | A | 6.8 | 1.8 |  |  |
| 83121-18-0 | teflubenzuron | IGR | >100 |  | 0.19* | 0.31* |
| 79538-32-2 | tefluthrin | I | 0.28 | 1.9 |  |  |
| 3383-96-8 | temephos | I | 1.6 |  |  |  |
| 13071-79-9 | terbufos | I | 4.1 | 4.1 |  |  |
| 886-50-0 | terbutryn | I | 0.16 | 1.22 |  |  |
| 22248-79-9 | tetrachlorvinphos | F | 1.5 |  |  |  |
| 112281-77-3 | tetraconazole | F | 63 | >130 |  |  |
| 116-29-0 | tetradifon | A | 1250 |  |  |  |
| 7696-12-0 | tetramethrin | I | 0.16 |  |  |  |
| 111988-49-9 | thiacloprid | I | 36 | 17 |  |  |
| 153719-23-4 | thiamethoxam | I | 0.025 | 0.005 |  |  |
| 130000-40-7 | thifluzamide | F | >100 | 51 |  |  |
| 31895-21-3 | thiocyclam | I |  | 12 |  |  |
| 59669-26-0 | thiodicarb | I | 12 |  |  |  |
| 640-15-3 | thiometon | I |  | 0.56 |  |  |
| 23564-05-8 | thiophanate-methyl | F | >100 | >100 |  |  |
| 137-26-8 | thiram | F | 74 | >100 |  |  |
| 57018-04-9 | tolclofos-methyl | F | >100 |  |  |  |
| 731-27-1 | tolylfluanid | F | >196 | >197 |  |  |
| 66841-25-6 | tralomethrin | I | 0.11 |  |  |  |
| 43121-43-3 | triadimefon | F | 50 |  |  |  |
| 112143-82-5 | triazamate | I | 27 | 41 |  |  |
| 72459-58-6 | triazoxide | F | >200 | >225 |  |  |
| 52-68-6 | trichlorfon | I | 18 |  | 568 |  |
| 81412-43-3 | tridemorph | F |  | >200 |  |  |
| 141517-21-7 | trifloxystrobin | F | >200 | >200 |  |  |
| 99387-89-0 | triflumizole | F | 140 |  |  |  |
| 26644-46-2 | triforine | F | >100 | >100 |  |  |
| 131983-72-7 | triticonazole | F | 49 | >100 |  |  |
| 50471-44-8 | vinclozolin | F | 141 |  |  |  |
| 2655-14-3 | XMC | I | 0.53 | 0.095 |  |  |
| 52315-07-8 | zeta-cypermethrin | I | 0.002 | 0.044 |  |  |
| 12122-67-7 | zineb | F | 7.1 | >100 |  |  |
| 137-30-4 | ziram | F | 86 | 447 |  |  |
| 156052-68-5 | zoxamide | F | >100 |  |  |  |
| 160791-64-0 | ZXI 8901 | I | 0.96 |  |  |  |

^1^ Sources: Pesticide Manual (2009), ECOTOX and AGRITOX databases

^2^ A = acaricide; B = biocide; F = fungicide; I = insecticide; IGR = insect growth regulator; IR = insecticide repellent; IS = synergist

* Chronic toxicity for 77 days exposure [3]
