# Supplementary material for: Pesticide Residues and Bees – A Risk Assessment
Source: PLoS One. 2014 Apr 9;9(4):e94482. doi: 10.1371/journal.pone.0094482 (PMC3981812; doi:10.1371/journal.pone.0094482)
Supplement: Table S3 — Estimated average and maximum daily doses (ng bee−1) of pesticide residues ingested by bees – herbicides excluded. (DOC) [file pone.0094482.s003.doc]

**Table S3.** Estimated average and maximum daily doses (ng bee^-1^) of pesticide residues ingested by bees – herbicides excluded.

| Chemical | *Apis mellifera* | | | | | | *Bombus* spp.^4^ | | | | | |
| --- | --- | --- | --- | --- | --- | --- | --- | --- | --- | --- | --- | --- |
|  | Worker larvae^1^ | | Nurse^2^ | | Nectar forager^3^ | | Worker larvae | | Nurse | | Nectar forager | |
|  | Average | Max | Average | Max | Average | Max | Average | Max | Average | Max | Average | Max |
| acephate | 0.100 | 1.682 | 0.591 | 1.060 | 0.000 | 4.170 | 0.499 | 8.390 | 2.949 | 5.284 | 0.000 | 20.800 |
| acetamiprid | 0.073 | 0.532 | 0.020 | 0.871 | 0.192 | 1.067 | 0.362 | 2.652 | 0.097 | 4.344 | 0.960 | 5.320 |
| acrinathrin (total) | 0.161 | 70.323 | 0.954 | 5.688 | 0.000 | 192.480 | 0.805 | 350.736 | 4.757 | 28.367 | 0.000 | 960.000 |
| aldicarb (total) | 0.014 | 1.476 | 0.085 | 8.724 | 0.000 | 0.000 | 0.071 | 7.364 | 0.422 | 43.513 | 0.000 | 0.000 |
| amitraz (total) | 0.064 | 1.229 | 0.380 | 7.261 | 0.000 | 0.000 | 0.320 | 6.128 | 1.893 | 36.212 | 0.000 | 0.000 |
| azoxystrobin | 0.008 | 0.233 | 0.050 | 0.696 | 0.000 | 0.321 | 0.042 | 1.164 | 0.248 | 3.469 | 0.000 | 1.600 |
| bendiocarb | 0.000 | 0.000 | 0.000 | 0.000 | 0.000 | 0.000 | 0.000 | 0.000 | 0.000 | 0.000 | 0.000 | 0.000 |
| beta-cyfluthrin | 0.263 | 0.407 | 0.014 | 0.221 | 0.722 | 1.027 | 1.309 | 2.032 | 0.071 | 1.102 | 3.600 | 5.120 |
| bifenthrin | 0.002 | 0.101 | 0.014 | 0.085 | 0.000 | 0.241 | 0.012 | 0.504 | 0.071 | 0.421 | 0.000 | 1.200 |
| bitertanol | 0.000 | 0.003 | 0.000 | 0.000 | 0.000 | 0.008 | 0.000 | 0.014 | 0.000 | 0.000 | 0.000 | 0.040 |
| boscalid | 0.025 | 1.058 | 0.146 | 6.253 | 0.000 | 0.000 | 0.123 | 5.278 | 0.728 | 31.187 | 0.000 | 0.000 |
| bromopropylate | 0.463 | 7.277 | 0.174 | 1.164 | 1.203 | 19.649 | 2.309 | 36.296 | 0.867 | 5.803 | 6.000 | 98.000 |
| captan (total) | 0.903 | 11.948 | 5.335 | 67.360 | 0.000 | 1.524 | 4.503 | 59.593 | 26.609 | 335.958 | 0.000 | 7.600 |
| carbaryl | 0.741 | 2.369 | 0.383 | 6.825 | 1.877 | 3.368 | 3.696 | 11.814 | 1.908 | 34.040 | 9.360 | 16.800 |
| carbendazim | 0.057 | 2.760 | 0.338 | 11.700 | 0.000 | 2.165 | 0.285 | 13.767 | 1.686 | 58.354 | 0.000 | 10.800 |
| carbofuran (total) | 1.474 | 18.792 | 0.079 | 0.894 | 4.054 | 51.729 | 7.353 | 93.724 | 0.394 | 4.458 | 20.220 | 258.000 |
| chlordane | 0.019 | 0.025 | 0.114 | 0.150 | 0.000 | 0.000 | 0.096 | 0.126 | 0.567 | 0.746 | 0.000 | 0.000 |
| chlorfenapyr | 0.001 | 0.002 | 0.008 | 0.009 | 0.000 | 0.000 | 0.007 | 0.008 | 0.042 | 0.045 | 0.000 | 0.000 |
| chlorfenvinphos | 0.045 | 0.468 | 0.239 | 2.730 | 0.014 | 0.016 | 0.226 | 2.333 | 1.191 | 13.616 | 0.068 | 0.080 |
| chlorothalonil | 1.171 | 109.247 | 5.215 | 642.850 | 0.802 | 1.267 | 5.843 | 544.871 | 26.008 | 3206.234 | 4.000 | 6.320 |
| chlorpyrifos | 0.148 | 1.347 | 0.212 | 5.395 | 0.310 | 1.203 | 0.736 | 6.716 | 1.057 | 26.908 | 1.546 | 6.000 |
| clothianidin | 0.065 | 0.337 | 0.061 | 0.268 | 0.152 | 0.810 | 0.325 | 1.682 | 0.305 | 1.336 | 0.760 | 4.040 |
| coumaphos (total) | 3.190 | 64.887 | 0.834 | 38.461 | 8.461 | 162.004 | 15.910 | 323.624 | 4.160 | 191.823 | 42.197 | 808.000 |
| cypermethrin | 0.539 | 2.729 | 0.090 | 0.416 | 1.452 | 7.378 | 2.686 | 13.612 | 0.450 | 2.075 | 7.243 | 36.800 |
| cyproconazole | 0.008 | 0.008 | 0.049 | 0.049 | 0.000 | 0.000 | 0.041 | 0.041 | 0.243 | 0.243 | 0.000 | 0.000 |
| cyprodinil | 0.015 | 0.378 | 0.086 | 2.236 | 0.000 | 0.000 | 0.073 | 1.887 | 0.429 | 11.152 | 0.000 | 0.000 |
| DDT (total) | 1.313 | 19.137 | 0.203 | 0.715 | 3.548 | 52.772 | 6.549 | 95.447 | 1.011 | 3.566 | 17.698 | 263.200 |
| deltamethrin | 0.162 | 0.293 | 0.167 | 0.592 | 0.371 | 0.534 | 0.809 | 1.460 | 0.833 | 2.950 | 1.853 | 2.665 |
| diazinon | 0.501 | 1.058 | 0.055 | 0.273 | 1.363 | 2.807 | 2.497 | 5.275 | 0.275 | 1.362 | 6.800 | 14.000 |
| dichlorvos | 0.239 | 0.395 | 0.044 | 0.061 | 0.642 | 1.067 | 1.190 | 1.969 | 0.220 | 0.305 | 3.200 | 5.320 |
| dicloran | 0.000 | 0.058 | 0.000 | 0.000 | 0.000 | 0.160 | 0.000 | 0.288 | 0.000 | 0.000 | 0.000 | 0.800 |
| dicofol | 0.078 | 2.758 | 0.081 | 0.930 | 0.179 | 7.218 | 0.389 | 13.757 | 0.404 | 4.636 | 0.890 | 36.000 |
| difenoconazole | 0.076 | 0.262 | 0.451 | 1.392 | 0.000 | 0.072 | 0.380 | 1.304 | 2.247 | 6.941 | 0.000 | 0.360 |
| diflubenzuron | 0.088 | 0.141 | 0.518 | 0.832 | 0.000 | 0.000 | 0.437 | 0.702 | 2.582 | 4.150 | 0.000 | 0.000 |
| dimethoate | 0.141 | 0.255 | 0.015 | 0.027 | 0.384 | 0.695 | 0.703 | 1.272 | 0.075 | 0.136 | 1.917 | 3.467 |
| dimethomorph | 0.012 | 0.183 | 0.069 | 1.079 | 0.000 | 0.000 | 0.058 | 0.911 | 0.342 | 5.382 | 0.000 | 0.000 |
| dinotefuran | 0.444 | 0.809 | 0.294 | 1.093 | 1.095 | 1.732 | 2.216 | 4.036 | 1.469 | 5.450 | 5.460 | 8.640 |
| endosulfan (total) | 0.674 | 4.278 | 0.502 | 20.670 | 1.634 | 2.165 | 3.361 | 21.338 | 2.506 | 103.092 | 8.149 | 10.800 |
| esfenvalerate | 0.004 | 0.066 | 0.021 | 0.390 | 0.000 | 0.000 | 0.018 | 0.329 | 0.105 | 1.945 | 0.000 | 0.000 |
| ethion | 0.000 | 0.000 | 0.000 | 0.000 | 0.000 | 0.000 | 0.000 | 0.000 | 0.000 | 0.000 | 0.000 | 0.000 |
| etoxazole | 0.000 | 0.029 | 0.000 | 0.000 | 0.000 | 0.080 | 0.000 | 0.144 | 0.000 | 0.000 | 0.000 | 0.400 |
| famoxadone | 0.108 | 0.155 | 0.639 | 0.917 | 0.000 | 0.000 | 0.539 | 0.774 | 3.187 | 4.571 | 0.000 | 0.000 |
| fenamidone | 0.081 | 0.081 | 0.480 | 0.480 | 0.000 | 0.000 | 0.405 | 0.405 | 2.396 | 2.396 | 0.000 | 0.000 |
| fenbuconazole | 0.110 | 0.436 | 0.653 | 2.574 | 0.000 | 0.000 | 0.551 | 2.173 | 3.256 | 12.838 | 0.000 | 0.000 |
| fenhexamid | 0.056 | 0.200 | 0.333 | 1.183 | 0.000 | 0.000 | 0.281 | 0.999 | 1.663 | 5.900 | 0.000 | 0.000 |
| fenitrothion | 0.006 | 0.008 | 0.036 | 0.046 | 0.000 | 0.000 | 0.030 | 0.038 | 0.178 | 0.227 | 0.000 | 0.000 |
| fenpyroximate | 0.031 | 0.125 | 0.185 | 0.741 | 0.000 | 0.000 | 0.156 | 0.625 | 0.921 | 3.696 | 0.000 | 0.000 |
| fenthion | 0.045 | 0.217 | 0.267 | 1.281 | 0.000 | 0.000 | 0.225 | 1.081 | 1.332 | 6.387 | 0.000 | 0.000 |
| fenvalerate | 0.020 | 0.020 | 0.000 | 0.000 | 0.056 | 0.056 | 0.100 | 0.100 | 0.000 | 0.000 | 0.278 | 0.278 |
| fipronil (total) | 0.002 | 0.032 | 0.010 | 0.189 | 0.000 | 0.000 | 0.009 | 0.159 | 0.051 | 0.940 | 0.000 | 0.000 |
| flumethrin | 0.000 | 0.029 | 0.000 | 0.000 | 0.000 | 0.080 | 0.000 | 0.144 | 0.000 | 0.000 | 0.000 | 0.400 |
| fluoxastrobin | 0.000 | 0.000 | 0.000 | 0.000 | 0.000 | 0.000 | 0.000 | 0.000 | 0.000 | 0.000 | 0.000 | 0.000 |
| flusilazole | 0.016 | 0.079 | 0.095 | 0.462 | 0.000 | 0.002 | 0.080 | 0.394 | 0.473 | 2.302 | 0.000 | 0.012 |
| flutolanil | 0.000 | 0.000 | 0.000 | 0.000 | 0.000 | 0.000 | 0.000 | 0.000 | 0.000 | 0.000 | 0.000 | 0.000 |
| gamma-HCH (lindane) | 5.109 | 124.584 | 0.049 | 0.150 | 14.155 | 345.662 | 25.482 | 621.368 | 0.246 | 0.746 | 70.598 | 1724.000 |
| heptenophos | 2.312 | 6.647 | 0.000 | 0.000 | 6.416 | 18.446 | 11.531 | 33.152 | 0.000 | 0.000 | 32.000 | 92.000 |
| hexaconazole | 0.060 | 0.117 | 0.356 | 0.689 | 0.000 | 0.000 | 0.300 | 0.582 | 1.773 | 3.436 | 0.000 | 0.000 |
| imazalil | 0.001 | 0.001 | 0.007 | 0.007 | 0.000 | 0.000 | 0.005 | 0.005 | 0.032 | 0.032 | 0.000 | 0.000 |
| imidacloprid (total) | 0.194 | 3.107 | 0.128 | 5.928 | 0.478 | 5.839 | 0.967 | 15.498 | 0.638 | 29.566 | 2.384 | 29.124 |
| indoxacarb | 0.119 | 0.459 | 0.704 | 2.711 | 0.000 | 0.000 | 0.594 | 2.288 | 3.513 | 13.519 | 0.000 | 0.000 |
| iprodione | 0.004 | 0.011 | 0.023 | 0.065 | 0.000 | 0.000 | 0.019 | 0.055 | 0.114 | 0.324 | 0.000 | 0.000 |
| lambda-cyhalothrin | 0.028 | 0.062 | 0.046 | 0.235 | 0.056 | 0.061 | 0.140 | 0.309 | 0.231 | 1.174 | 0.280 | 0.306 |
| malathion | 2.851 | 7.100 | 0.111 | 0.455 | 7.859 | 19.489 | 14.218 | 35.410 | 0.553 | 2.269 | 39.195 | 97.200 |
| metalaxyl | 0.016 | 0.042 | 0.096 | 0.246 | 0.000 | 0.000 | 0.081 | 0.208 | 0.477 | 1.229 | 0.000 | 0.000 |
| methamidophos | 0.024 | 0.024 | 0.143 | 0.143 | 0.000 | 0.000 | 0.121 | 0.121 | 0.713 | 0.713 | 0.000 | 0.000 |
| methidathion | 1.157 | 2.002 | 0.006 | 0.215 | 3.208 | 5.454 | 5.771 | 9.983 | 0.029 | 1.070 | 16.000 | 27.200 |
| methiocarb | 0.435 | 0.782 | 0.009 | 0.009 | 1.203 | 2.165 | 2.170 | 3.899 | 0.045 | 0.045 | 6.000 | 10.800 |
| methomyl | 0.011 | 0.026 | 0.067 | 0.156 | 0.000 | 0.000 | 0.057 | 0.132 | 0.334 | 0.778 | 0.000 | 0.000 |
| methoxychlor | 0.000 | 17.138 | 0.000 | 0.000 | 0.000 | 47.559 | 0.000 | 85.475 | 0.000 | 0.000 | 0.000 | 237.200 |
| methoxyfenozide | 0.003 | 0.228 | 0.019 | 0.832 | 0.000 | 0.241 | 0.016 | 1.135 | 0.094 | 4.150 | 0.000 | 1.200 |
| mevinphos | 0.000 | 0.000 | 0.000 | 0.000 | 0.000 | 0.000 | 0.000 | 0.000 | 0.000 | 0.000 | 0.000 | 0.000 |
| myclobutanil | 0.176 | 4.609 | 1.038 | 27.235 | 0.000 | 0.000 | 0.876 | 22.988 | 5.175 | 135.835 | 0.000 | 0.000 |
| oxamyl | 0.034 | 0.054 | 0.201 | 0.319 | 0.000 | 0.000 | 0.170 | 0.269 | 1.003 | 1.589 | 0.000 | 0.000 |
| parathion | 0.016 | 0.021 | 0.092 | 0.125 | 0.000 | 0.000 | 0.077 | 0.105 | 0.457 | 0.622 | 0.000 | 0.000 |
| parathion methyl | 0.316 | 1.472 | 0.161 | 0.161 | 0.802 | 4.010 | 1.577 | 7.343 | 0.804 | 0.804 | 4.000 | 20.000 |
| penconazole | 0.019 | 0.139 | 0.114 | 0.819 | 0.000 | 0.000 | 0.097 | 0.691 | 0.571 | 4.085 | 0.000 | 0.000 |
| permethrin | 0.012 | 0.882 | 0.068 | 0.598 | 0.000 | 2.165 | 0.058 | 4.397 | 0.340 | 2.983 | 0.000 | 10.800 |
| phenothrin | 0.092 | 0.092 | 0.545 | 0.545 | 0.000 | 0.000 | 0.460 | 0.460 | 2.720 | 2.720 | 0.000 | 0.000 |
| phorate | 0.006 | 0.026 | 0.000 | 0.000 | 0.018 | 0.071 | 0.032 | 0.128 | 0.000 | 0.000 | 0.088 | 0.356 |
| phosalone | 0.034 | 0.034 | 0.203 | 0.203 | 0.000 | 0.000 | 0.172 | 0.172 | 1.015 | 1.015 | 0.000 | 0.000 |
| phosmet | 0.373 | 18.212 | 2.205 | 107.614 | 0.000 | 0.000 | 1.861 | 90.831 | 10.999 | 536.728 | 0.000 | 0.000 |
| piperonyl butoxide | 0.000 | 0.289 | 0.000 | 0.000 | 0.000 | 0.802 | 0.000 | 1.441 | 0.000 | 0.000 | 0.000 | 4.000 |
| pirimicarb | 1.098 | 2.052 | 0.000 | 0.000 | 3.048 | 5.694 | 5.477 | 10.234 | 0.000 | 0.000 | 15.200 | 28.400 |
| pirimiphos ethyl | 0.549 | 0.636 | 0.000 | 0.000 | 1.524 | 1.764 | 2.739 | 3.171 | 0.000 | 0.000 | 7.600 | 8.800 |
| prallethrin | 0.007 | 0.008 | 0.042 | 0.049 | 0.000 | 0.000 | 0.036 | 0.042 | 0.211 | 0.246 | 0.000 | 0.000 |
| propiconazole | 0.006 | 0.397 | 0.036 | 2.347 | 0.000 | 0.000 | 0.030 | 1.981 | 0.178 | 11.703 | 0.000 | 0.000 |
| propoxur | 0.008 | 0.008 | 0.049 | 0.049 | 0.000 | 0.000 | 0.041 | 0.041 | 0.243 | 0.243 | 0.000 | 0.000 |
| pyraclostrobin | 0.028 | 0.783 | 0.166 | 1.723 | 0.000 | 1.363 | 0.140 | 3.904 | 0.826 | 8.591 | 0.000 | 6.800 |
| pyrazophos | 0.179 | 0.214 | 0.000 | 0.000 | 0.497 | 0.593 | 0.894 | 1.067 | 0.000 | 0.000 | 2.480 | 2.960 |
| pyrethrins | 0.000 | 0.068 | 0.003 | 0.403 | 0.000 | 0.000 | 0.002 | 0.340 | 0.013 | 2.010 | 0.000 | 0.000 |
| pyridaben | 0.021 | 0.029 | 0.122 | 0.173 | 0.000 | 0.000 | 0.103 | 0.146 | 0.609 | 0.862 | 0.000 | 0.000 |
| pyrimethanil | 0.016 | 0.207 | 0.092 | 0.540 | 0.000 | 0.321 | 0.078 | 1.032 | 0.460 | 2.691 | 0.000 | 1.600 |
| pyriproxyfen | 0.000 | 0.000 | 0.000 | 0.000 | 0.000 | 0.000 | 0.000 | 0.000 | 0.000 | 0.000 | 0.000 | 0.000 |
| quinalphos | 0.277 | 0.297 | 0.000 | 0.000 | 0.769 | 0.824 | 1.382 | 1.480 | 0.000 | 0.000 | 3.836 | 4.108 |
| quintozene | 0.000 | 0.000 | 0.000 | 0.000 | 0.000 | 0.000 | 0.000 | 0.000 | 0.000 | 0.000 | 0.000 | 0.000 |
| rotenone | 0.025 | 0.025 | 0.150 | 0.150 | 0.000 | 0.000 | 0.126 | 0.126 | 0.746 | 0.746 | 0.000 | 0.000 |
| spirodiclofen | 0.000 | 0.000 | 0.000 | 0.000 | 0.000 | 0.000 | 0.000 | 0.000 | 0.000 | 0.000 | 0.000 | 0.000 |
| spiromesifen | 0.038 | 0.069 | 0.223 | 0.410 | 0.000 | 0.000 | 0.188 | 0.346 | 1.110 | 2.042 | 0.000 | 0.000 |
| tau-fluvalinate | 0.595 | 24.612 | 0.802 | 17.355 | 1.275 | 60.150 | 2.969 | 122.753 | 3.999 | 86.559 | 6.360 | 300.000 |
| tebuconazole | 0.134 | 0.181 | 0.107 | 0.216 | 0.321 | 0.401 | 0.667 | 0.903 | 0.535 | 1.076 | 1.600 | 2.000 |
| tebufenozide | 0.001 | 0.064 | 0.007 | 0.377 | 0.000 | 0.000 | 0.006 | 0.318 | 0.036 | 1.880 | 0.000 | 0.000 |
| tefluthrin | 0.000 | 0.000 | 0.000 | 0.000 | 0.000 | 0.000 | 0.000 | 0.000 | 0.000 | 0.000 | 0.000 | 0.000 |
| temephos | 0.208 | 0.234 | 0.000 | 0.000 | 0.577 | 0.650 | 1.038 | 1.168 | 0.000 | 0.000 | 2.880 | 3.240 |
| tetradifon | 0.421 | 0.944 | 0.096 | 1.580 | 1.123 | 1.877 | 2.099 | 4.706 | 0.477 | 7.878 | 5.600 | 9.360 |
| tetramethrin | 0.007 | 0.007 | 0.040 | 0.040 | 0.000 | 0.000 | 0.033 | 0.033 | 0.198 | 0.198 | 0.000 | 0.000 |
| thiacloprid | 0.270 | 7.137 | 0.488 | 6.514 | 0.521 | 16.746 | 1.349 | 35.595 | 2.434 | 32.490 | 2.600 | 83.520 |
| thiamethoxam | 0.217 | 0.631 | 0.188 | 0.826 | 0.513 | 1.363 | 1.081 | 3.147 | 0.937 | 4.117 | 2.560 | 6.800 |
| thiophanate-methyl | 0.122 | 1.554 | 0.721 | 9.185 | 0.000 | 0.000 | 0.608 | 7.752 | 3.595 | 45.808 | 0.000 | 0.000 |
| triadimefon | 0.000 | 0.000 | 0.000 | 0.000 | 0.000 | 0.000 | 0.000 | 0.000 | 0.000 | 0.000 | 0.000 | 0.000 |
| trichlorfon | 0.015 | 0.015 | 0.091 | 0.091 | 0.000 | 0.000 | 0.077 | 0.077 | 0.454 | 0.454 | 0.000 | 0.000 |
| trifloxystrobin | 0.015 | 0.299 | 0.091 | 1.716 | 0.000 | 0.024 | 0.077 | 1.492 | 0.452 | 8.559 | 0.000 | 0.120 |
| vinclozolin | 2.164 | 3.823 | 0.013 | 0.046 | 5.999 | 10.586 | 10.793 | 19.065 | 0.066 | 0.227 | 29.920 | 52.800 |

1 Worker larvae intake = 1.1 mg pollen day^-1^ plus 28.9 mg honey day^-1^ [76]

2 Nurse intake = 6.5 mg pollen day^-1^ [76]

3 Nectar forager intake = 80.2 mg nectar or honey day^-1^ [76]

4 Same proportions as *Apis m.* but scaled for average consumption of *Bombus* spp. (see File SI)
