# Supplementary material for: Pesticide Residues and Bees – A Risk Assessment
Source: PLoS One. 2014 Apr 9;9(4):e94482. doi: 10.1371/journal.pone.0094482 (PMC3981812; doi:10.1371/journal.pone.0094482)
Supplement: File SI — Estimation of doses and risks by contact and dietary exposure. (DOCX) [file pone.0094482.s004.docx]

**Supporting Information**

**Estimation of doses and risks by contact and dietary exposure**

***Conversion from LC50 to LD50***

Some toxicity data are reported in the databases as oral LC50s, in which case the corresponding 48-h oral LD50s were calculated based on the daily consumption of sugar (either as honey or nectar) by the bees as follows


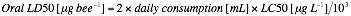
 (S1)

For worker honey bees, an average daily consumption of 32.4 mg of sugar (~25.5 ml) was used based on previous studies [[1](#_ENREF_1)]. In laboratory experiments, worker bumble bees consume on average 400 mg (~315 ml) syrup [[2](#_ENREF_2)]. Topical LC50s were converted to LD50s in a similar way, assuming an average 1 µL droplet was applied to honey bees [[3](#_ENREF_3)] and 4 µL to bumble bees [[4](#_ENREF_4)].


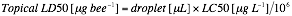
 (S2)

***Risk by contact exposure***

In the case of exposure by contact with pollen, topical LD50s shown in Table S2 were used to calculate the risk for a worker bee that comes in contact with 1 g of contaminated pollen per day, as follows


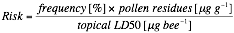
 (S3)

***Risk by dietary exposure***

In a similar way, dietary doses for larvae and worker bees were estimated using the consumption rates of pollen and honey or nectar described by Rortais et al. [[1](#_ENREF_1)]. Since honey appears to be concentrated nectar, these authors estimate daily consumption rates on the basis of total sugar intake (mg) in either case. Bee larvae are typically fed a mixture of pollen and honey for 5 to 6.5 days, depending on whether the larvae will develop as a worker or a drone, respectively. Among the worker bees there is a variety of consumption figures depending on their role in the colony (see Table 1). Worker bees usually start as nurses and then transition to foraging duties at about 3 weeks of age [[5](#_ENREF_5),[6](#_ENREF_6)].

Estimates of daily intake of residues for each of the three bee types and species were calculated prior to their risk as


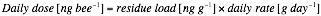
 (S4)

and they are shown in Table S3 for average and maximum loads respectively. Consequently, the risk of each bee type ingesting contaminated pollen and nectar during their entire life span (days) was calculated as


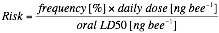
 (S5)

Data used in this study are found in Tables S1, S2 and S3.

**References**

1. Rortais A, Arnold G, Halm MP, Touffet-Briens F (2005) Modes of honeybees exposure to systemic insecticides: estimated amounts of contaminated pollen and nectar consumed by different categories of bees. Apidologie 36: 71-83.

2. Laycock I, Lenthall K, Barratt A, Cresswell J (2012) Effects of imidacloprid, a neonicotinoid pesticide, on reproduction in worker bumble bees (*Bombus terrestris*). Ecotoxicology 21: 1937-1945.

3. Iwasa T, Motoyama N, Ambrose JT, Roe RM (2004) Mechanism for the differential toxicity of neonicotinoid insecticides in the honey bee, *Apis mellifera*. Crop Protection 23: 371-378.

4. Helson BV, Barber KN, Kingsbury PD (1994) Laboratory toxicology of six forestry insecticides to four species of bee (Hymenoptera: Apoidea). Archives of Environmental Contamination and Toxicology 27: 107-114.

5. Goblirsch M, Huang ZY, Spivak M (2013) Physiological and behavioral changes in honey bees (*Apis mellifera*) induced by *Nosema ceranae* infection. PLoS One 8: e58165.

6. Winston ML (1987) The biology of the honey bee. Cambridge, MA: Harvard University Press.
